# Lipid Metabolism and Ferroptosis in Cancer: Regulatory Mechanisms, Alternative Splicing, and Therapeutic Implications

**DOI:** 10.3390/ijms27209244

**Published:** 2026-10-18

**Authors:** Lucia Maria Procopciuc, Adriana Corina Hangan, Roxana Liana Lucaciu

**Affiliations:** 1 Department of Medical Biochemistry, “Iuliu-Hațieganu” University of Medicine and Pharmacy, 400349 Cluj-Napoca, Romania; lprocopciuc@umfcluj.ro; 2 Department of Inorganic Chemistry, Faculty of Pharmacy, “Iuliu-Hațieganu” University of Medicine and Pharmacy, 400012 Cluj-Napoca, Romania; 3 Department of Pharmaceutical Biochemistry and Clinical Laboratory, Faculty of Pharmacy, “Iuliu-Hațieganu” University of Medicine and Pharmacy, 400349 Cluj-Napoca, Romania; liana.lucaciu@umfcluj.ro

**Keywords:** ferroptosis, alternative splicing, lipid metabolism, cancer, isoform switching, lipid peroxidation, therapeutic resistance, precision oncology

## Abstract

Ferroptosis is an iron-dependent form of regulated cell death driven by phospholipid peroxidation and is increasingly recognized as an important vulnerability in cancer. Its sensitivity is determined by the interplay between membrane lipid composition, iron availability, oxidative stress, and antioxidant defense. Cancer cells can adapt to these pressures by altering the balance between polyunsaturated and monounsaturated fatty acids, thereby shifting toward either ferroptosis sensitivity or resistance. Emerging evidence indicates that alternative splicing provides an additional regulatory layer by generating functionally distinct isoforms of genes involved in cystine transport, iron metabolism, oxidative stress, antioxidant protection, and lipid remodeling. Functionally validated splice events affecting the cystine/glutamate antiporter subunit, transferrin receptor, spermidine/spermine acetyltransferase 1, SCL/TAL1 interrupting locus protein, and cluster of differentiation 44 demonstrate that isoform switching can influence ferroptotic susceptibility independently of total gene expression. This review integrates current knowledge on the biochemical basis of ferroptosis, the lipid determinants of ferroptosis sensitivity, and splicing-dependent mechanisms that shape vulnerability and resistance in cancer. Therapeutic strategies targeting ferroptosis defense systems, lipid metabolism, and alternative splicing are also discussed. Finally, we highlight the potential of integrating long-read ribonucleic acid sequencing, lipidomics, proteomics, and functional ferroptosis assays to develop multidimensional vulnerability signatures for patient stratification and precision cancer therapy.

## 1. Introduction

Ferroptosis has emerged as a distinct form of regulated cell death with major implications for cancer biology and therapy. Initially defined as an iron-dependent, non-apoptotic form of cell death, ferroptosis is now recognized as a process driven by the uncontrolled accumulation of oxidized phospholipids and the collapse of cellular mechanisms that normally restrain lipid peroxidation [1,2]. In contrast to apoptosis, which is primarily executed through caspase-dependent proteolytic programs, ferroptosis is fundamentally linked to cellular metabolism and redox homeostasis, with iron availability, membrane lipid composition, reactive oxygen species (ROS), and antioxidant capacity acting together to determine whether cells survive or undergo ferroptotic death [2,3]. This distinctive metabolic basis has generated considerable interest in ferroptosis as a potentially exploitable vulnerability in malignant cells, especially in tumors that are poorly responsive to conventional treatments or have acquired resistance to apoptosis-based therapies [3,4].

Cancer cells undergo extensive metabolic adaptation in order to sustain proliferation, membrane synthesis, redox balance, and survival in nutrient- and oxygen-variable environments [5]. Increased iron demand, elevated basal oxidative stress, enhanced lipid synthesis and remodeling, and dependence on antioxidant defense pathways can place malignant cells close to a critical threshold of lipid oxidative damage [4,6]. At the same time, however, tumor cells can reinforce ferroptosis-suppressive pathways and remodel their metabolism in ways that allow them to survive persistent oxidative stress. Therefore, ferroptosis in cancer should not be viewed simply as a tumor-suppressive death mechanism, but rather as the outcome of a dynamic balance between pro-ferroptotic metabolic pressure and adaptive anti-ferroptotic defenses [6,7].

Among the metabolic processes involved, lipid metabolism plays a particularly important role because phospholipid peroxidation represents the biochemical execution step of ferroptosis [8]. Cellular sensitivity is strongly influenced by the abundance and composition of membrane phospholipids, especially those containing polyunsaturated fatty acids (PUFAs), which are highly susceptible to oxidative attack because of their multiple bis-allylic hydrogen atoms [3,8]. Thus, ferroptosis susceptibility is not determined merely by the amount of total cellular lipid, but by the qualitative organization of the lipidome and by the enzymatic pathways that control fatty-acid activation, desaturation, incorporation into phospholipids, storage, and mobilization [5,8].

Several lipid metabolic enzymes have consequently emerged as important determinants of ferroptotic sensitivity. Acyl-CoA synthetase long-chain family member 4 (ACSL4) promotes activation of polyunsaturated fatty acids such as arachidonic and adrenic acids, while lysophosphatidylcholine acyltransferase 3 (LPCAT3) contributes to their incorporation into membrane phospholipids, thereby enriching the cellular pool of oxidizable lipid substrates [3,4]. By contrast, metabolic programs that increase the abundance or utilization of monounsaturated fatty acids (MUFAs) can protect membranes from oxidative damage and reduce susceptibility to ferroptosis [8,9]. Stearoyl-CoA desaturase 1 (SCD1), for example, has been linked to tumor resistance to ferroptosis by increasing fatty-acid desaturation and supporting a more oxidation-resistant lipid environment [9].

Lipid storage and mobilization add another level of complexity to this relationship. Lipid droplets, once regarded primarily as passive repositories of neutral lipids, are now considered dynamic metabolic organelles that regulate lipid availability, oxidative stress, and cellular adaptation. By temporarily sequestering fatty acids away from membrane phospholipids or releasing them under metabolic stress, lipid droplets can alter the availability of substrates for lipid peroxidation. In cancer, this buffering function may contribute to adaptation to fluctuating nutrient conditions, oxidative stress, and anticancer therapy [10].

The presence of oxidizable membrane lipids, however, is not sufficient by itself to trigger ferroptosis. Cancer cells possess several antioxidant systems that continuously neutralize lipid peroxides and thereby oppose ferroptotic death. The best characterized of these is the cystine/glutamate antiporter system Xc^−^ (system Xc^−^)–glutathione (GSH)–glutathione peroxidase 4 (GPX4) axis. The cystine/glutamate antiporter system Xc^−^, whose light-chain subunit is encoded by solute carrier family 7 member 11 (SLC7A11), supports intracellular cysteine availability and glutathione synthesis, whereas GPX4 uses reduced glutathione to convert phospholipid hydroperoxides into less reactive lipid alcohols [2,11]. Consequently, increased SLC7A11 activity, preservation of glutathione pools, or sustained GPX4 function can shift cancer cells toward ferroptosis resistance, while disruption of this axis can dramatically increase susceptibility to lethal lipid peroxidation [3,12].

Additional GPX4-independent defense mechanisms have broadened the concept of ferroptosis resistance. Ferroptosis suppressor protein 1 (FSP1) limits lipid peroxidation by maintaining reduced coenzyme Q species capable of trapping lipid radicals at cellular membranes [13]. Other protective systems include the dihydroorotate dehydrogenase (DHODH)–CoQ axis in mitochondria and the GTP cyclohydrolase 1 (GCH1)–tetrahydrobiopterin pathway, which can suppress ferroptosis through antioxidant activity and lipid remodeling [7,13].

This balance between a peroxidation-prone lipid environment and antioxidant protection has important consequences for cancer progression and treatment response.

Conversely, pharmacological or genetic disruption of GPX4, SLC7A11, SCD1, or related ferroptosis-defense mechanisms can sensitize selected tumor models to ferroptotic death and enhance the effects of other anticancer treatments [12,14]. These observations provide a compelling rationale for identifying the molecular mechanisms that determine whether cancer cells occupy a ferroptosis-sensitive or ferroptosis-resistant metabolic state.

While most studies of ferroptosis have focused on metabolic enzymes, redox regulators, and signaling pathways, RNA processing is increasingly recognized as another mechanism capable of reshaping cancer-cell metabolism. Alternative splicing (AS) enables a single precursor messenger RNA to generate multiple transcript isoforms through differential selection of exons or splice sites, thereby expanding transcriptomic and proteomic diversity [15]. The resulting isoforms may differ in enzymatic activity, subcellular localization, protein stability, interaction partners, regulatory domains, or response to signaling cues [15,16]. In cancer, alternative splicing is frequently altered through changes in the expression or activity of splicing factors and RNA-binding proteins, mutations affecting components of the spliceosome, alterations in cis-regulatory elements, and oncogenic signaling pathways that impinge on RNA processing [16,17]. Aberrant splicing has consequently been linked to tumor-cell proliferation, apoptosis evasion, invasion, metastasis, metabolic adaptation, and treatment resistance [16,17,18].

The relationship between alternative splicing and cancer metabolism is particularly relevant to the present context. Increasing evidence indicates that cancer-associated splicing programs can generate metabolically distinct protein isoforms and redirect the activity of pathways involved in nutrient utilization, biosynthesis, energy production, and redox regulation [17,18,19]. Conversely, metabolic conditions and nutrient-sensing pathways can affect the activity of splicing factors and spliceosomal components, suggesting bidirectional communication between RNA processing and cellular metabolism [19]. This emerging concept raises the possibility that some of the metabolic determinants of ferroptosis may be controlled not only at the level of gene expression or protein abundance, but also through isoform selection.

Direct experimental evidence supporting this possibility has begun to emerge. In breast cancer cells, Wang et al. recently demonstrated that fragile X mental retardation protein (FMRP) interacts with the splicing factor heterogeneous nuclear ribonucleoprotein M (hnRNPM) and regulates an exon-skipping event within the SLC7A11 transcript. The resulting SLC7A11-S splice variant promoted ferroptosis resistance, while the FMRP–hnRNPM–SLC7A11 axis was associated with more aggressive disease features and poorer clinical outcome [20]. This finding is particularly important because it demonstrates that the functional state of a major ferroptosis-defense gene can depend on alternative splicing rather than simply on total transcript abundance.

Despite substantial progress in understanding ferroptosis, lipid remodeling, and cancer-associated alternative splicing individually, their intersection remains incompletely characterized. Recent reviews have extensively examined lipid metabolism as a determinant of ferroptosis sensitivity [8] and have separately summarized the contribution of aberrant splicing to cancer metabolism and malignant progression [16,17,18,19]. However, comparatively little attention has been devoted to the possibility that alternative splicing may reshape lipid composition, lipid-peroxidation susceptibility, or ferroptosis-defense pathways in a coordinated manner. This represents an important knowledge gap because isoform-level regulation could explain biological differences that are not apparent from conventional gene-expression analyses and could reveal tumor-specific vulnerabilities that would otherwise remain undetected.

Accordingly, this review aims to integrate current evidence on alternative splicing, lipid metabolism, and ferroptosis in cancer, with particular emphasis on how isoform-level regulation may determine ferroptosis vulnerability or resistance. We first summarize the biochemical basis of ferroptosis and the major antioxidant systems that restrain lipid peroxidation, followed by an analysis of lipid-remodeling pathways that establish pro-ferroptotic or ferroptosis-resistant membrane states. We then focus on emerging evidence that alternative splicing and RNA-binding proteins modulate ferroptosis-related and lipid-metabolic genes, thereby altering cystine metabolism, iron availability, antioxidant defense, lipid composition, and susceptibility to lipid peroxidation. Finally, we discuss the biological and therapeutic implications of these mechanisms for tumor progression, treatment resistance, and patient stratification.

The distinctive contribution of this review is the proposal of an isoform-centered framework linking alternative splicing to lipid metabolic control of ferroptosis in cancer. By integrating validated splice events with lipid remodeling, iron metabolism, antioxidant defense, and ferroptosis-related signaling, we highlight alternative splicing as a regulatory mechanism capable of modifying ferroptotic susceptibility independently of overall gene expression. This perspective further identifies the splicing–lipid metabolism–ferroptosis axis as a potential source of mechanistic biomarkers and therapeutic vulnerabilities that may not be captured by conventional gene-level analyses.

## 2. Literature Search Strategy

A narrative literature search was performed to identify relevant studies addressing ferroptosis, lipid metabolism, alternative splicing, and their intersection in cancer. PubMed, Scopus, and Web of Science were searched using combinations of terms including “ferroptosis”, “cancer”, “lipid metabolism”, “polyunsaturated fatty acids”, “monounsaturated fatty acids”, “ACSL4”, “ACSL3”, “SCD1”, “GPX4”, “SLC7A11”, “FSP1”, “alternative splicing”, “splice isoform”, “splicing factor”, and “RNA-binding protein”. Particular attention was given to studies investigating functionally validated splice events affecting ferroptosis-related genes or lipid-metabolic pathways.

Priority was given to original research articles, mechanistic studies, and recent reviews published mainly between 2019 and 2026, while seminal earlier studies were included when necessary to describe the biochemical foundations of ferroptosis or key regulatory mechanisms. Additional references were identified through manual screening of the reference lists of relevant articles. Studies were selected on the basis of their mechanistic relevance to ferroptosis sensitivity or resistance, lipid remodeling, antioxidant defense, alternative splicing, and therapeutic response in cancer. Because this article is a narrative rather than a systematic review, no formal meta-analysis or quantitative evidence synthesis was performed.

## 3. Ferroptosis in Cancer: Biochemical Basis and Metabolic Vulnerability

### 3.1. Core Biochemical Mechanisms of Ferroptosis

Ferroptosis is a regulated form of cell death characterized by the iron-dependent accumulation of lipid peroxides in cellular membranes. Unlike death programs primarily executed through proteolytic cascades or pore-forming proteins, ferroptosis arises from the convergence of disturbances in iron homeostasis, lipid composition, ROS generation, and antioxidant capacity [21,22]. Its defining biochemical event is the uncontrolled peroxidation of membrane phospholipids, which progressively alters membrane properties and ultimately results in loss of cellular integrity [21,23].

Iron is essential to this process because redox-active iron facilitates reactions that initiate and propagate lipid oxidation. Intracellular iron homeostasis involves tightly coordinated uptake, storage, utilization, and export, whereas the metabolically accessible labile iron pool provides Fe^2+^ capable of participating in redox reactions [24,25]. Through Fenton chemistry, Fe^2+^ reacts with hydrogen peroxide to generate highly reactive hydroxyl radicals that can initiate oxidative damage, including the abstraction of hydrogen atoms from susceptible membrane lipids [25,26]. Iron can also support lipid peroxidation through iron-containing oxidoreductases and lipoxygenase-associated processes, indicating that its contribution to ferroptosis extends beyond classical Fenton chemistry [26,27].

Changes in iron trafficking therefore substantially influence ferroptotic susceptibility. Transferrin receptor-mediated uptake increases intracellular iron availability, whereas ferritin sequestration limits the pool of redox-active iron. Conversely, degradation of ferritin through ferritinophagy releases stored iron and can promote ferroptosis by expanding the labile iron pool [24,28].

Although ROS generation contributes to ferroptotic stress, generalized oxidative stress alone does not define ferroptosis. The critical substrates are oxidation-prone membrane phospholipids, particularly those containing PUFAs [21,29]. Lipid peroxyl radicals can subsequently oxidize neighboring fatty-acid chains, creating a self-propagating lipid-peroxidation chain reaction [26,29].

Phosphatidylethanolamines containing arachidonic or adrenic acid were among the first lipid species identified as preferentially oxidized during ferroptosis [30]. Their abundance is metabolically regulated. ACSL4 activates long-chain PUFAs and promotes the generation of PUFA-CoA derivatives that can subsequently be incorporated into membrane phospholipids, thereby increasing the cellular pool of ferroptosis-sensitive substrates [31].

Lipid peroxidation can proceed through both non-enzymatic and enzymatic mechanisms. Iron-derived radical chemistry can initiate spontaneous phospholipid autoxidation, whereas lipoxygenases and other oxidoreductases can facilitate selective lipid oxidation under specific cellular conditions [26,27]. Cytochrome P450 oxidoreductase (POR), for example, has been identified as an important contributor to phospholipid peroxidation and ferroptosis in several cellular contexts [32].

Once lipid hydroperoxides accumulate beyond the capacity of cellular defense systems, their effects extend beyond chemical oxidation of individual phospholipids. Lipid peroxidation modifies membrane fluidity, curvature, thickness, permeability, and interactions between lipids and membrane proteins [21,33]. These changes progressively destabilize cellular membranes and interfere with ion homeostasis, establishing the conditions required for ferroptotic execution [23,33].

Rather than representing an instantaneous rupture caused solely by the physical accumulation of oxidized lipids, ferroptotic execution appears to involve changes in membrane tension and cation permeability, followed by osmotic imbalance and progressive plasma-membrane dysfunction [33,34]. When these protective processes are overwhelmed, plasma-membrane integrity is irreversibly lost and ferroptotic cell death ensues [21,33,34].

Ferroptosis is distinguishable from other major forms of regulated cell death by both its biochemical requirements and its execution machinery. Apoptosis is typically mediated by intrinsic or extrinsic signaling pathways converging on caspase activation, particularly the executioner caspases−3 and −7. Ferroptosis is caspase-independent and instead requires uncontrolled phospholipid peroxidation. Necroptosis depends predominantly on receptor-interacting serine/threonine-protein kinase 3 (RIPK3)-dependent activation of mixed lineage kinase domain-like protein (MLKL), whose oligomerization disrupts the plasma membrane, whereas ferroptosis does not require the canonical RIPK3–MLKL pathway. Pyroptosis, in turn, is mediated by inflammatory caspases and gasdermin pore formation, a mechanism fundamentally distinct from the oxidative membrane injury characteristic of ferroptosis [35].

Morphological features also differ from those of classical apoptosis. Mitochondrial shrinkage, increased membrane density, and loss of cristae have frequently been described, although mitochondrial alterations are neither universally present nor sufficient to identify ferroptosis [1]. Consequently, ferroptosis should not be diagnosed on morphology alone. Experimental identification generally requires complementary evidence of lipid peroxidation, iron dependence, and rescue by established ferroptosis-suppressing interventions [1,21].

The core biochemical architecture of ferroptosis can therefore be reduced to three interconnected requirements: redox-active iron, oxidation-prone membrane phospholipids, and insufficient control of lipid peroxide accumulation [21,22]. These antioxidant mechanisms, which enable cancer cells to suppress lipid peroxidation and acquire ferroptosis resistance, are discussed in the following section.

### 3.2. Ferroptosis Defense Systems

Because lipid peroxidation can arise continuously from cellular oxidative metabolism, survival requires efficient mechanisms capable of preventing the accumulation and propagation of phospholipid hydroperoxides. Ferroptosis therefore differs conceptually from many other forms of regulated cell death in that its suppression depends on constitutively active metabolic defense systems rather than solely on inhibition of a dedicated death-execution pathway [21,36]. Among the currently recognized anti-ferroptotic systems, the system Xc^−^–SLC7A11–GSH–GPX4 axis represents the best-characterized defense mechanism, while the FSP1–CoQ10 pathway provides an important parallel protective system. Additional GPX4-independent mechanisms, including DHODH–ubiquinol (CoQH2) and GTP cyclohydrolase 1–tetrahydrobiopterin (GCH1–BH4), further increase the metabolic flexibility with which cancer cells can suppress lipid peroxidation [3,21,36].

The system Xc^−^ cystine/glutamate antiporter constitutes an important upstream component of the canonical ferroptosis-defense pathway. This heterodimeric transporter consists of the light-chain subunit SLC7A11 (xCT), which confers substrate specificity, and the heavy-chain subunit solute carrier family 3 member 2 (SLC3A2), which supports membrane localization and transporter stability [37]. System Xc^−^ imports extracellular cystine in exchange for intracellular glutamate. Once inside the cell, cystine is rapidly reduced to cysteine, an amino acid required for the synthesis of glutathione [37,38].

Glutathione is synthesized from glutamate, cysteine, and glycine through sequential enzymatic reactions involving glutamate-cysteine ligase and glutathione synthetase. Its reduced form, GSH, represents one of the major intracellular thiol antioxidants and serves as an essential reducing cofactor for GPX4 [21,39]. This mechanism underlies the ferroptosis-inducing activity of compounds such as erastin and related inhibitors of system Xc^−^, which reduce cystine availability, deplete intracellular GSH, and indirectly impair GPX4 activity [1,38]. Conversely, increased SLC7A11 expression can reinforce antioxidant capacity and confer resistance to oxidative and ferroptotic stress in several cancer contexts [37,40].

At the downstream end of this axis, GPX4 represents a central enzymatic suppressor of ferroptosis. Using GSH as an electron donor, GPX4 converts phospholipid hydroperoxides (PL-OOH) into their corresponding non-toxic phospholipid alcohols (PL-OH), while GSH is oxidized to glutathione disulfide (GSSG). This reaction interrupts the propagation of lipid-peroxidation chains and prevents oxidized phospholipids from reaching levels incompatible with membrane integrity [39,41].

The pivotal role of GPX4 in ferroptosis was established by studies demonstrating that either direct GPX4 inhibition or depletion of cellular glutathione is sufficient to induce ferroptotic death in susceptible cancer cells [41]. tumor cell states become particularly dependent on GPX4 for survival because their lipid composition and oxidative environment continuously generate ferroptotic pressure [41,42].

The SLC7A11–GSH–GPX4 pathway is therefore best viewed as a coordinated metabolic axis rather than as a collection of independent molecules. Disruption at any of these levels can lower the ferroptotic threshold, whereas activation of the pathway can promote resistance [21,37]. In cancer, regulation of SLC7A11 and GPX4 is integrated with oncogenic signaling, stress-response pathways, nutrient sensing, and transcriptional control, allowing malignant cells to dynamically adapt their ferroptosis-defense capacity to environmental conditions [40,43].

The discovery of FSP1 demonstrated, however, that GPX4 is not the only major enzymatic barrier to ferroptosis. FSP1 functions predominantly as an NAD(P)H-dependent oxidoreductase and, following N-terminal myristoylation, associates with cellular membranes where it reduces ubiquinone, or coenzyme Q10 (CoQ10), to its antioxidant form CoQH2 [44,45].

CoQH2 acts as a lipophilic radical-trapping antioxidant, intercepting lipid peroxyl radicals and terminating lipid-peroxidation chain reactions before extensive membrane damage develops [44,46]. Thus, whereas GPX4 enzymatically reduces pre-existing phospholipid hydroperoxides, the FSP1–CoQ10 system suppresses the propagation of lipid radical reactions through regeneration of reduced CoQ. Importantly, FSP1 can maintain ferroptosis resistance even when GPX4 activity is compromised, explaining why GPX4 inhibition alone does not necessarily induce equivalent ferroptotic responses in all cancer cells [44,45].

The identification of FSP1 also established reduced nicotinamide adenine dinucleotide (phosphate) (NAD(P)H) and CoQ metabolism as important components of cellular ferroptosis defense. Reduced pyridine nucleotides provide the reducing power required for continuous regeneration of membrane-associated antioxidants, linking central metabolism to ferroptosis resistance [46,47].

A further layer of protection operates within mitochondria through DHODH. DHODH is located in the inner mitochondrial membrane and normally catalyzes the oxidation of dihydroorotate to orotate during de novo pyrimidine synthesis. In doing so, it transfers electrons to CoQ and generates reduced CoQH2 [48]. Mao et al. demonstrated that mitochondrial DHODH can suppress ferroptosis by reducing CoQ and limiting mitochondrial lipid peroxidation, particularly in cells with relatively low mitochondrial GPX4 activity [48]. Its importance appears to depend on cellular metabolic context, and inhibition of DHODH can enhance ferroptotic sensitivity in selected tumor models, especially when other antioxidant defenses are insufficient.

Another GPX4-independent mechanism is provided by the GCH1–BH4 pathway. GCH1 catalyzes the rate-limiting step in BH4 biosynthesis, and genome-wide screening identified GCH1 as a potent suppressor of ferroptosis. BH4 and its related metabolites can function as radical-trapping antioxidants, while activation of the GCH1–BH4 pathway also produces changes in cellular lipid composition that reduce the susceptibility of selected phospholipid species to peroxidation [49]. Regeneration of BH4 from its oxidized forms, including through dihydrofolate reductase-dependent reactions, further supports the persistence of this antioxidant system [49,50].

This redundancy has important implications for cancer biology. Tumor cells capable of activating several ferroptosis-defense systems may tolerate substantial lipid oxidative stress and acquire a highly resistant phenotype, whereas tumors dependent predominantly on one pathway may display a therapeutically exploitable vulnerability [21,22]. As discussed in the following section, this balance is profoundly influenced by the lipid composition of cancer cells and by the metabolic pathways controlling the synthesis, remodeling, storage, and utilization of fatty acids and phospholipids.

**Figure 1:**
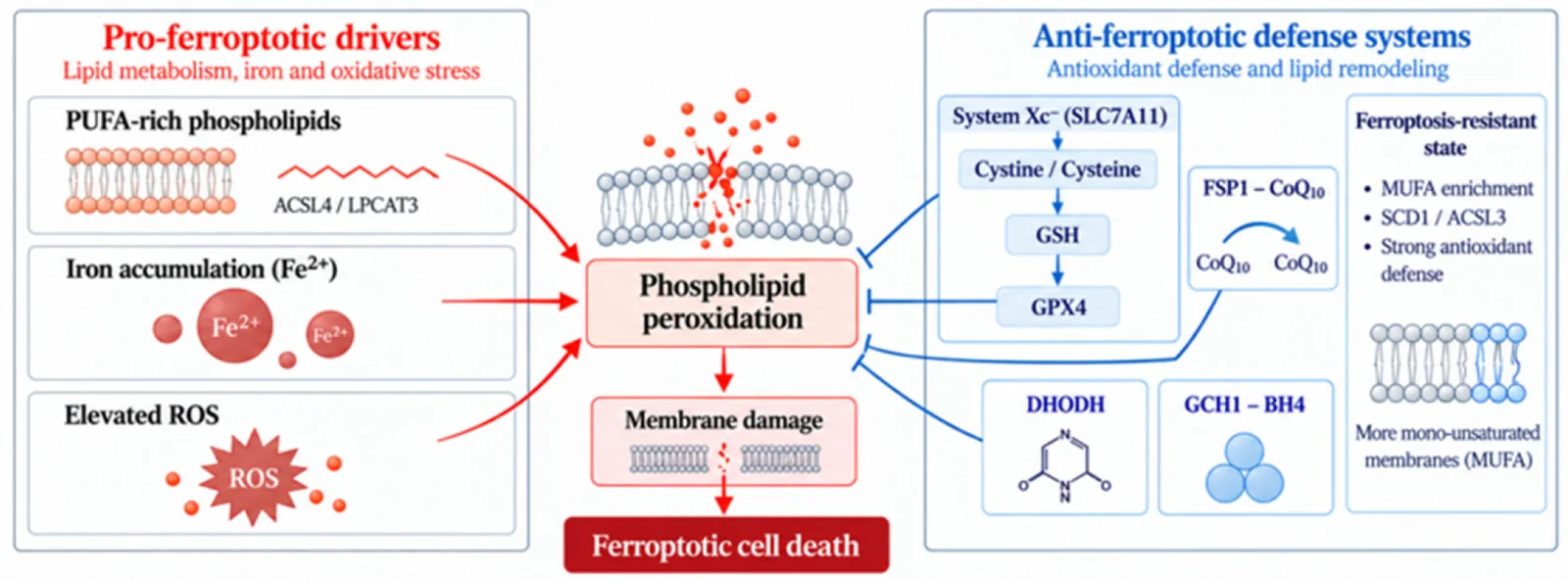
Biochemical determinants and defense systems regulating ferroptosis in cancer. PUFA-rich phospholipids, increased intracellular iron, and elevated reactive oxygen species promote phospholipid peroxidation, membrane damage, and ultimately ferroptotic cell death. In contrast, the SLC7A11–GSH–GPX4 axis, FSP1–CoQ system, DHODH, and GCH1–BH4 pathway suppress lipid peroxidation and support ferroptosis resistance. MUFA enrichment, supported by SCD1 and ACSL3, further reduces membrane susceptibility to peroxidative damage. Together, these opposing processes determine the balance between ferroptosis sensitivity and resistance in cancer cells. Abbreviations: ACSL3, acyl-CoA synthetase long-chain family member 3; ACSL4, acyl-CoA synthetase long-chain family member 4; BH4, tetrahydrobiopterin; CoQ, coenzyme Q; CoQH2, reduced coenzyme Q; DHODH, dihydroorotate dehydrogenase; FSP1, ferroptosis suppressor protein 1; GCH1, GTP cyclohydrolase 1; GPX4, glutathione peroxidase 4; GSH, reduced glutathione; LPCAT3, lysophosphatidylcholine acyltransferase 3; MUFA, monounsaturated fatty acid; PUFA, polyunsaturated fatty acid; ROS, reactive oxygen species; SCD1, stearoyl-CoA desaturase 1; SLC7A11, solute carrier family 7 member 11.

The major biochemical drivers of ferroptosis and the principal cellular defense systems that counteract phospholipid peroxidation are summarized in Figure 1.

### 3.3. Why Cancer Cells Are Vulnerable to Ferroptosis

Cancer cells undergo profound metabolic adaptations to sustain continuous proliferation, biomass production, survival under nutrient limitation, and resistance to environmental stress. Although these adaptations provide a selective advantage during tumor development, they can also generate metabolic liabilities that increase susceptibility to ferroptosis [4,51]. Together, these features can position cancer cells close to the threshold at which lipid-peroxidation damage becomes irreversible, creating a potentially exploitable ferroptosis vulnerability [4,52].

One important determinant is the increased requirement for iron. Rapidly proliferating cancer cells need iron for DNA synthesis, mitochondrial metabolism, electron-transport reactions, ribonucleotide reductase activity, and the function of numerous iron-dependent enzymes [53,54]. Many tumors therefore increase iron acquisition while reducing iron export or modifying ferritin storage, resulting in expansion of intracellular iron pools [53]. This iron-dependent phenotype, sometimes referred to as iron addiction, supports malignant growth but carries an intrinsic risk because excess redox-active Fe^2+^ can promote ROS generation and lipid peroxidation [54,55].

Cancer cells also frequently exist under chronically elevated oxidative stress. Increased metabolic activity, mitochondrial ROS production, oncogenic signaling, inflammatory mediators, hypoxia-reoxygenation cycles, and altered reduced nicotinamide adenine dinucleotide phosphate (NADPH) metabolism can all contribute to higher basal ROS levels than those observed in many non-malignant cells [56,57]. Ferroptosis exploits this fragile equilibrium because even a modest additional increase in lipid oxidative stress or a reduction in antioxidant capacity may be sufficient to drive phospholipid peroxidation beyond a survivable level [4,58].

A further source of vulnerability arises from the exceptionally high demand for membrane synthesis. Proliferating tumor cells must continuously generate new plasma and organellar membranes, requiring enhanced synthesis, uptake, and remodeling of fatty acids, phospholipids, and cholesterol [59]. Importantly, when newly synthesized or remodeled membranes become enriched in PUFA-containing phospholipids, the abundance of substrates susceptible to peroxidation also increases [8,30]. The precise balance between PUFA-rich and MUFA-rich membrane species therefore becomes a major determinant of whether high membrane synthesis promotes ferroptosis susceptibility or instead supports resistance [8].

Tumor cells can increase de novo fatty-acid synthesis, uptake exogenous lipids, alter fatty-acid desaturation, enhance lipid storage, or modify phospholipid remodeling according to tumor type and microenvironmental conditions [59,60]. Enrichment in PUFA-containing phospholipids creates a ferroptosis-prone lipidome, whereas increased MUFA production, altered lipid trafficking, or sequestration of fatty acids in lipid droplets may protect against peroxidative damage [8,61]. This dual role is particularly relevant in cancer because lipid metabolic pathways are frequently rewired during progression and therapeutic adaptation [8,60].

Paradoxically, the same oxidative and lipid metabolic pressures that increase ferroptosis susceptibility also force cancer cells to become highly dependent on antioxidant defense systems. SLC7A11-mediated cystine uptake, glutathione synthesis, GPX4 activity, FSP1-dependent CoQ reduction, and nuclear factor erythroid 2-related factor 2 (NRF2)-regulated antioxidant programs can become essential for maintaining membrane integrity under conditions of persistent oxidative stress [37,55]. In such cells, inhibition of GPX4, depletion of GSH, disruption of SLC7A11 activity, or interference with complementary antioxidant pathways can produce a disproportionately strong effect because the malignant cell has limited residual capacity to compensate [4,37].

This concept is particularly evident in therapy-resistant tumor-cell states. Several cancer models with mesenchymal, drug-resistant, or highly invasive phenotypes show increased dependence on lipid-peroxide detoxification systems, including GPX4 [42]. In these contexts, treatment resistance does not necessarily eliminate vulnerability; rather, adaptation to conventional therapy may create a new metabolic dependency that can potentially be exploited through ferroptosis induction.

Importantly, ferroptosis susceptibility is highly heterogeneous. Not all cancers accumulate iron to the same extent, generate equivalent amounts of ROS, contain similar membrane lipid profiles, or rely on the same antioxidant pathways [4,8]. Tumor lineage, differentiation state, oncogenic mutations, metabolic environment, nutrient availability, and prior treatment exposure can all modify the balance between ferroptosis-promoting and ferroptosis-suppressing processes [4,52]. Consequently, ferroptosis vulnerability should be regarded as a context-dependent metabolic phenotype rather than a universal property of malignant cells.

Among the factors determining where this balance is established, the composition and remodeling of the cellular lipidome are particularly important and are therefore examined in detail in the following section.

## 4. Lipid Metabolism as a Determinant of Ferroptosis Sensitivity

### 4.1. PUFA and Ferroptosis-Promoting Lipid Remodeling

The susceptibility of cancer cells to ferroptosis is strongly determined by the molecular composition of their membrane phospholipids. However, free PUFAs alone do not efficiently drive ferroptosis. Their pro-ferroptotic potential becomes particularly relevant after they are activated and incorporated into membrane phospholipids, where they form oxidation-prone substrates that can undergo radical propagation and generate phospholipid hydroperoxides [3,30,31].

Arachidonic acid (AA; 20:4) and adrenic acid (AdA; 22:4) are among the best-characterized PUFAs associated with ferroptotic membrane damage. Early lipidomic studies demonstrated that phosphatidylethanolamine species containing AA or AdA become preferentially oxidized during ferroptosis and can act as major death signals when their hydroperoxide derivatives accumulate [30]. More recent studies have expanded this concept to other PUFA-containing phospholipids and have shown that both the number and position of double bonds, as well as the phospholipid head group and subcellular membrane localization, can influence susceptibility to lipid peroxidation [3,62].

The incorporation of PUFAs into phospholipids occurs through coordinated lipid-remodeling pathways, commonly associated with the Lands’ cycle. Recent work has therefore placed phospholipid remodeling at the center of ferroptosis regulation, because changing the balance between PUFA-containing and saturated or monounsaturated phospholipid species can shift cells toward either ferroptosis sensitivity or resistance [62].

A major enzyme controlling this process is ACSL4. ACSL4 catalyzes the ATP-dependent conversion of long-chain fatty acids into their corresponding acyl-CoA derivatives and displays a marked preference for PUFAs such as AA and AdA acids [31,63]. Genetic and pharmacological studies established ACSL4 as a major determinant of ferroptosis sensitivity, because ACSL4 deficiency decreases the abundance of PUFA-containing membrane phospholipids and confers resistance to ferroptotic stimuli [31].

The role of ACSL4 is particularly relevant in cancer because its biological effects are context-dependent. ACSL4 can support tumor growth, migration, or metabolic adaptation in some malignancies, while at the same time creating a lipid environment that renders cells more susceptible to ferroptosis. When sufficient antioxidant defenses are present, ACSL4-driven PUFA activation may support lipid metabolism and membrane remodeling without causing lethal damage. However, when lipid-peroxide detoxification becomes insufficient, the same ACSL4-dependent phospholipid composition can facilitate rapid ferroptotic execution [63,64].

Following PUFA activation by ACSL4, lysophosphatidylcholine acyltransferase 3 (LPCAT3) contributes to the incorporation of activated PUFA acyl chains into membrane phospholipids. LPCAT3 belongs to the lysophospholipid acyltransferase family and participates in phospholipid remodeling by reacylating lysophospholipid intermediates with fatty acyl-CoA substrates [65]. Experimental disruption of LPCAT3 has been shown to reduce PUFA incorporation into membrane phospholipids and diminish ferroptosis sensitivity, supporting its role as an important downstream partner of ACSL4 in generating a pro-ferroptotic membrane landscape [62,65].

Once incorporated into phospholipids, PUFAs can undergo enzymatic or non-enzymatic oxidation. Lipoxygenases, POR, and iron-dependent radical reactions can generate phospholipid hydroperoxides from PUFA-containing membrane lipids [3,32]. Importantly, the rate of lipid peroxidation depends on both the abundance of susceptible substrates and the efficiency of antioxidant countermeasures, meaning that the same PUFA-rich membrane composition may remain tolerated in one cellular context but become lethal in another [3,66].

Rapidly proliferating cells require continuous membrane synthesis and extensive phospholipid remodeling, increasing the demand for fatty-acid activation and incorporation into structural lipids [60]. Accordingly, tumors with high ACSL4 activity or an elevated PUFA-phospholipid signature may, under appropriate conditions, display increased sensitivity to ferroptosis-inducing therapies [63,64].

At the same time, the relationship between ACSL4, LPCAT3, and ferroptosis should not be considered absolute. Recent lipidomic studies have demonstrated that additional acyl-CoA synthetases, lysophospholipid acyltransferases, phospholipases, and lipid-transfer proteins can influence ferroptosis by modifying phospholipid composition [62]. Moreover, di-PUFA phospholipids and alternative PUFA-containing lipid classes have emerged as additional substrates capable of promoting ferroptosis in specific contexts [62].

Overall, PUFA-containing phospholipids constitute a major biochemical substrate for ferroptotic lipid peroxidation, while ACSL4 and LPCAT3 play key roles in generating and maintaining these oxidation-sensitive membrane species. Importantly, this pro-ferroptotic PUFA program is counterbalanced by lipid metabolic pathways that enrich membranes with more oxidation-resistant monounsaturated fatty acids. These anti-ferroptotic mechanisms are discussed in the following section.

### 4.2. MUFA and Ferroptosis Resistance

In contrast to PUFAs, MUFAs generally reduce cellular susceptibility to ferroptosis because they are considerably less prone to lipid peroxidation. Their protective effect depends not only on their abundance but also on their incorporation into membrane phospholipids, where they can partially replace oxidation-prone PUFA-containing species and thereby decrease the availability of substrates required for propagation of lipid-peroxidation reactions [8,61]. Experimental evidence has shown that exogenous MUFAs such as oleic acid and palmitoleic acid suppress lipid ROS accumulation and protect cells from ferroptosis induced by system Xc^−^ inhibition or GPX4 inactivation [61].

Importantly, MUFAs do not appear to protect primarily by acting as classical radical-scavenging antioxidants. Instead, their anti-ferroptotic effect arises predominantly through lipid remodeling, whereby MUFA-containing phospholipids displace or dilute PUFA-containing species in membrane compartments [61,67].

A central enzyme controlling endogenous MUFA production is SCD1. SCD1 is an endoplasmic-reticulum-associated Δ9 desaturase that introduces a cis double bond into saturated fatty acyl-CoAs, converting palmitoyl-CoA and stearoyl-CoA primarily into palmitoleoyl-CoA and oleoyl-CoA, respectively [68]. Its ability to increase cellular MUFA availability also provides an important mechanism of protection against ferroptotic lipid damage.

Increased SCD1 activity raises the MUFA content of membrane lipids and can simultaneously reduce the relative abundance of PUFA-containing phospholipids available for peroxidation [68,69]. As a result, inhibition or depletion of SCD1 often increases lipid ROS accumulation and sensitizes cancer cells to ferroptosis-inducing conditions. Recent pan-cancer analyses further support the concept that SCD1-dependent desaturation constitutes a recurrent metabolic program through which diverse oncogenic contexts converge on ferroptosis resistance [69].

The biological importance of this pathway is particularly evident in tumor cells subjected to therapeutic stress. In breast cancer models, enhanced “de novo” lipogenesis and increased incorporation of MUFAs into cellular membranes were recently shown to protect dormant disseminated tumor cells from ferroptosis and facilitate metastatic persistence. Genetic suppression of ACSL3 or pharmacological interference with “de novo” lipogenesis increased lipid peroxidation and triggered ferroptotic death in these cells, indicating that MUFA-dependent membrane remodeling can support long-term survival of residual tumor-cell populations [70]. Similar principles have been observed after radiotherapy, where surviving cancer cells displayed increased oleic and palmitoleic acid levels together with reduced lipid peroxidation, and exogenous supplementation with these MUFAs further increased resistance to ferroptosis [71].

ACSL3 plays a key role in this process by converting free long-chain fatty acids into fatty acyl-CoA derivatives that can enter phospholipid and neutral-lipid synthesis pathways. Magtanong et al. demonstrated that the ferroptosis-suppressive effect of exogenous MUFAs requires ACSL3 activity. Loss of ACSL3 prevented oleic acid-mediated protection, increased plasma-membrane lipid oxidation, and restored ferroptosis susceptibility, establishing ACSL3 as a critical mediator of the MUFA-induced ferroptosis-resistant state [61]. Importantly, this protection was independent of lipid-droplet formation in the experimental system, indicating that direct remodeling of membrane phospholipids rather than neutral-lipid storage was sufficient to account for the anti-ferroptotic effect.

Whereas ACSL4 preferentially activates PUFAs and promotes the formation of ferroptosis-sensitive phospholipid species, ACSL3 facilitates the utilization of MUFAs and favors a membrane environment that is more resistant to peroxidation [31,61].

Recent cancer-specific studies further reinforce the relevance of ACSL3. In cholangiocarcinoma, increased ACSL3 expression was associated with resistance to the GPX4 inhibitor RAS-selective lethal 3 (RSL3) and with higher levels of MUFA-containing phospholipids. ACSL3 silencing restored ferroptosis sensitivity, supporting a direct role for this enzyme in maintaining an anti-ferroptotic lipid profile. Clinically, higher ACSL3 expression was also associated with adverse prognostic features, suggesting that the same metabolic program that protects tumor cells from ferroptosis may contribute to disease aggressiveness [72].

In several tumor models, disruption of either MUFA synthesis or utilization is sufficient to shift this balance back toward ferroptosis sensitivity [31,69,70,71,72].

Importantly, MUFA-mediated ferroptosis resistance is not absolute and can be overcome by altering the PUFA/MUFA balance. Recent work in high-risk neuroblastoma demonstrated that pharmacological or genetic inhibition of SCD1 restored ferroptosis sensitivity in resistant cells, while supplementation with AA further enhanced ferroptotic death by increasing the relative abundance of PUFA substrates [73]. These findings support the concept that ferroptosis susceptibility depends on the relative composition of the membrane lipidome rather than on the absolute abundance of a single fatty-acid class.

Overall, MUFA synthesis and incorporation constitute a major lipid-based defense against ferroptosis in cancer. At the same time, dependence on the SCD1–ACSL3–MUFA axis may create a therapeutic vulnerability, because disruption of this protective lipid program can restore ferroptosis sensitivity in selected tumor contexts.

### 4.3. Lipid Synthesis, Storage and Mobilization

Beyond the relative abundance of PUFAs and MUFAs, ferroptosis sensitivity is shaped by the continuous flux of fatty acids between synthesis, storage, mobilization, and membrane incorporation. Cancer cells frequently increase both endogenous lipid synthesis and uptake from the extracellular environment in order to sustain proliferation, membrane biogenesis, signaling, and adaptation to metabolic stress [60,74]. Lipid synthesis may increase the availability of fatty acids for membrane remodeling, whereas sequestration of oxidation-prone fatty acids into neutral-lipid stores can temporarily protect them from peroxidation. Conversely, mobilization of stored lipids can release fatty acids that are subsequently incorporated into membrane phospholipids and thereby alter ferroptotic susceptibility [8,75].

“De novo” lipid synthesis is frequently enhanced in malignant cells and is primarily driven by the coordinated activity of ATP-citrate lyase, acetyl-CoA carboxylase, fatty-acid synthase (FASN), and desaturases such as SCD1 [59,60]. In the context of ferroptosis, however, the relevance of this pathway depends on the lipid species ultimately generated. In contrast, “de novo” lipid synthesis may also support phospholipid production and membrane expansion, increasing the capacity of cells to incorporate exogenous or endogenously generated PUFAs into oxidation-prone phospholipid species [36,74]. The functional consequence is therefore context-dependent and determined by the balance between fatty-acid saturation, desaturation, elongation, and phospholipid incorporation rather than by the overall rate of lipogenesis alone.

An important consequence of high lipid synthesis or uptake is the accumulation of excess fatty acids that cannot immediately be incorporated into membranes or oxidized for energy. These lipids are frequently esterified into neutral lipids, predominantly triacylglycerols and cholesteryl esters, and stored within lipid droplets (LDs). LDs consist of a hydrophobic neutral-lipid core surrounded by a phospholipid monolayer and associated proteins and are increasingly recognized as dynamic organelles involved in lipid trafficking, energy supply, redox regulation, and stress adaptation [10,75]. Their abundance is frequently increased in cancer cells, particularly under hypoxic, nutrient-stressed, or therapy-induced conditions, where they provide metabolic flexibility and contribute to tumor-cell survival [75,76].

In ferroptosis, LDs can function as regulators of the availability of peroxidation-prone fatty acids. One protective mechanism involves the sequestration of PUFAs into neutral lipids, thereby reducing their immediate incorporation into membrane phospholipids [8,77].

Recent experimental evidence provides direct support for this protective role. In PUFA-supplemented triple-negative breast cancer cells, inhibition of diacylglycerol acyltransferase (DGAT)-dependent lipid-droplet formation caused extensive redistribution of PUFAs from neutral-lipid stores into membrane glycerophospholipids. This remodeling increased membrane unsaturation, enhanced lipid peroxidation, and markedly increased ferroptosis sensitivity [77].

The relationship between lipid droplets and ferroptosis is nevertheless not uniformly protective. LDs are highly dynamic structures, and the fatty acids stored within them can be released through lipolysis or lipophagy when cells require energy or membrane substrates [75,78]. If the released fatty-acid pool contains substantial quantities of PUFAs, mobilization can potentially increase the supply of substrates available for phospholipid remodeling and lipid peroxidation [8,78]. Consequently, LDs may either suppress or facilitate ferroptosis depending on whether lipid storage or lipid mobilization predominates and on the chemical composition of the stored fatty acids.

Therefore, the effect of LDs on ferroptosis cannot be inferred from their abundance alone. Instead, their contribution depends on rates of lipid synthesis, DGAT-mediated esterification, lipolysis, lipophagy, and the trafficking of released fatty acids between cellular compartments [75,77].

Ultimately, the impact of synthesis, storage, and mobilization converges on phospholipid remodeling, because fatty acids must be incorporated into membrane phospholipids to influence ferroptotic execution directly. Remodeling pathways continuously exchange acyl chains between lysophospholipids and activated fatty-acyl-CoA pools, allowing cancer cells to modify membrane composition in response to metabolic conditions [36,64]. However, recent work indicates that ferroptotic sensitivity is governed by a broader network of acyl-CoA synthetases, acyltransferases, phospholipases, and lipid-transfer pathways that together determine the degree and topology of membrane unsaturation [36,79].

Phosphatidylcholine metabolism provides one example of this broader regulatory architecture. Alterations in phosphatidylcholine synthesis and acyl-chain remodeling can change membrane PUFA content and modify ferroptosis susceptibility, while extracellular lipid exchange between tumor cells and stromal cells may further reshape phospholipid composition [80]. This suggests that the ferroptosis-sensitive lipidome is not generated exclusively within the cancer cell but can also be influenced by metabolic interactions within the tumor microenvironment.

Lipid synthesis, storage, mobilization, and phospholipid remodeling form an interconnected metabolic network that determines the availability and cellular destination of oxidation-prone fatty acids. Ferroptosis sensitivity therefore reflects lipid flux rather than static lipid abundance, with cancer cells continuously redistributing fatty acids between protective storage pools and oxidation-prone membrane compartments. This dynamic lipid organization provides an important metabolic background for understanding how additional regulatory mechanisms, including alternative splicing, may shift tumor cells toward ferroptosis vulnerability or resistance.

The major lipid metabolic determinants that shift cancer cells toward ferroptosis sensitivity or resistance are summarized in Table 1.

## 5. Alternative Splicing as a Regulator of Ferroptosis in Cancer

### 5.1. Alternative Splicing of Ferroptosis-Related Genes

Alternative splicing provides cancer cells with a rapid mechanism for generating functionally distinct protein isoforms from the same gene and can therefore modify ferroptosis sensitivity without requiring changes in overall gene expression. This concept is particularly relevant for genes involved in cystine transport, iron uptake, antioxidant defense, and lipid-peroxide control, because relatively small functional differences between isoforms may profoundly alter the balance between ferroptosis vulnerability and resistance. Emerging studies now indicate that cancer-associated splice switches can regulate ferroptosis through several distinct mechanisms, including changes in transporter activity, intracellular iron availability, glutathione metabolism, GPX4 expression, and the accumulation of lipid peroxides [20,81,82].

One of the clearest examples involves SLC7A11, the light-chain subunit of system Xc^−^ and a major determinant of ferroptosis resistance. In breast cancer, Wang et al. demonstrated that FMRP interacts with the splicing factor hnRNPM and promotes an exon-skipping event within SLC7A11 pre-mRNA [20]. Nanopore isoform sequencing identified two principal transcripts, a longer SLC7A11-L isoform and an exon-4-skipped SLC7A11-S isoform. Increased production of SLC7A11-S was associated with reduced lipid ROS and malondialdehyde accumulation, higher intracellular glutathione levels, and increased resistance to ferroptotic stress. Conversely, disruption of either FMRP or hnRNPM shifted SLC7A11 splicing, increased lipid peroxidation, and sensitized breast cancer cells to ferroptosis [20].

The SLC7A11 splice switch appears to be relevant beyond breast cancer. A more recent study in prostate cancer identified circCNOT6L as an upstream regulator of SLC7A11 alternative splicing. CircCNOT6L, which was enriched in metastatic prostate cancer, facilitated serine/arginine-rich splicing factor 2 (SRSF2)-dependent processing of SLC7A11 pre-mRNA and promoted a ferroptosis-resistant phenotype. Suppression of circCNOT6L increased ferroptotic sensitivity, whereas combination of circCNOT6L silencing with the ferroptosis inducer erastin markedly reduced viability in prostate-cancer-derived organoids [81].

Alternative splicing can also regulate ferroptosis by altering iron acquisition. In endometrial cancer, circRAPGEF5 was shown to interact with the splicing regulator RNA binding fox-1 homolog 2 (RBFOX2) and interfere with its binding to transferrin receptor pre-mRNA (TFRC). This interaction promoted exclusion of exon 4 and increased production of a truncated TFRC-S isoform. The protein generated from this transcript failed to localize efficiently to the plasma membrane and consequently displayed impaired iron-import capacity. Reduced transferrin-mediated iron uptake decreased the intracellular labile iron pool and attenuated lipid-peroxide accumulation, thereby conferring ferroptosis resistance [82]. Functional gain- and loss-of-function experiments confirmed that manipulating RBFOX2 could reverse the effects of circRAPGEF5 on both TFRC splicing and ferroptotic susceptibility. This study therefore illustrates how alternative splicing can regulate ferroptosis upstream of lipid peroxidation by directly controlling the availability of redox-active iron.

Another mechanistically distinct example involves spermidine/spermine N1-acetyltransferase 1 (SAT1), an enzyme involved in polyamine catabolism that can promote lipid peroxidation and ferroptosis. SAT1 pre-mRNA contains an alternative exon harboring a premature termination codon, and exclusion of this exon is required for production of the functional SAT1 protein. In lung cancer cells, the spliceosomal RNA-binding protein RNF113A was shown to regulate this event during cisplatin treatment. Loss of ring finger protein 113A (RNF113A) favored generation of the coding SAT1 transcript, markedly increased SAT1 protein expression, enhanced lipid ROS accumulation, and promoted ferroptotic cell death [83]. RNF113A overexpression, which occurs in lung cancer and contributes to cisplatin resistance, therefore appears to protect cells in part by maintaining a SAT1 splicing pattern that limits the formation of functional pro-ferroptotic SAT1 protein.

Alternative splicing can additionally influence ferroptosis indirectly through regulators that modify both iron metabolism and antioxidant defense. An illustrative example is PCLAF (PCNA clamp-associated factor) in hepatitis B virus-associated hepatocellular carcinoma. Alternative splicing of PCLAF exon 3 produces distinct transcript variants, and HBV infection was shown to promote accumulation of the PCLAF tv1 isoform by downregulating the splicing factor SRSF2. Increased PCLAF tv1 reduced intracellular Fe^2+^ levels while simultaneously increasing GPX4 expression, thereby decreasing ferroptotic activity and contributing to sorafenib resistance. Restoration of SRSF2 expression or disruption of the PCLAF tv1 axis reversed these effects, indicating that a virus-driven splicing program can connect isoform selection directly to ferroptosis-dependent drug resistance [84].

Recent evidence further expands the spectrum of ferroptosis-related genes controlled by alternative splicing. In ovarian cancer, increased histone H3 lysine 18 lactylation was reported to stimulate expression of the splicing factor TRA2A (transformer 2 alpha homolog), which in turn altered alternative splicing of STIL (SCL/TAL1 interrupting locus) and promoted formation of the STIL-L isoform [85]. STIL-L suppressed ferroptosis through effects on iron metabolism and simultaneously reduced sensitivity to cisplatin. Genetic depletion of TRA2A restored ferroptotic susceptibility and enhanced cisplatin efficacy in tumor models, demonstrating how an epigenetic-metabolic signal can be transmitted through a splicing factor to generate a ferroptosis-resistant isoform [85].

A particularly interesting example reported in lung cancer involves CD44, a highly alternatively spliced cell-surface glycoprotein associated with stemness, invasion, and metastatic behavior. MEN1 was recently shown to promote ferroptosis by limiting inclusion of cluster of differentiation 44 (CD44) variable exons [86]. Mechanistically, menin 1 (MEN1) slowed RNA polymerase II elongation through regulation of polymerase-associated factor 1 (PAF1) release, thereby restricting production of CD44 variant isoforms. Loss of MEN1 increased accumulation of CD44 variants, particularly CD44v6-containing transcripts, reduced cytosolic and lipid ROS, increased GPX4 expression, and decreased sensitivity to erastin-induced ferroptosis. Interference with CD44v6 restored lipid peroxide accumulation and ferroptosis in MEN1-deficient lung cancer cells and reduced tumor growth and metastasis in experimental models [86].

An important implication of these findings is that the biological effect of alternative splicing cannot always be predicted from total gene expression. This is particularly relevant for transcriptomic studies that quantify genes without resolving isoform composition. Long-read RNA sequencing and isoform-specific analyses may therefore reveal ferroptosis-associated vulnerabilities that remain hidden in conventional short-read or gene-level expression datasets [20,82].

The available evidence also suggests that isoform switching can operate in both directions. Many cancer-associated splice programs, including SLC7A11-S, TFRC-S, PCLAF tv1, STIL-L, and CD44 variants, favor ferroptosis resistance and thereby support malignant progression or treatment resistance [20,81,82,83,84,85,86]. In contrast, splicing events that increase functional SAT1 expression can promote ferroptosis and sensitize tumor cells to oxidative damage [83]. Thus, alternative splicing should not be viewed inherently as an anti-ferroptotic process; rather, it functions as a regulatory switch capable of moving cancer cells toward either ferroptosis vulnerability or ferroptosis resistance depending on the isoform generated and the metabolic context.

These studies provide direct mechanistic evidence that alternative splicing contributes to the regulation of ferroptosis in cancer. Although the number of experimentally validated examples remains relatively limited compared with the extensive literature on transcriptional and post-translational ferroptosis regulation, the diversity of identified mechanisms is already substantial. The next question is whether alternative splicing also remodels the lipid-metabolic machinery itself, thereby modifying the availability and composition of the phospholipid substrates required for ferroptotic execution.

### 5.2. Alternative Splicing of Lipid-Metabolic Genes

Alternative splicing of lipid-metabolic genes represents a particularly attractive mechanism through which cancer cells could modify ferroptosis susceptibility. However, compared with the growing number of experimentally validated splice events affecting canonical ferroptosis-defense genes, direct evidence linking alternative splicing of lipid-metabolic genes to ferroptosis remains relatively limited. Current data therefore support an emerging rather than fully established regulatory layer, with several cancer-associated examples demonstrating profound effects on lipid metabolism but only a subset being directly connected to ferroptotic sensitivity [17,87].

#### 5.2.1. Candidate Mechanisms: ACSL4 and ACSL3 Splice Variant

The long-chain acyl-CoA synthetase (ACSL) family represents an obvious candidate for such regulation because these enzymes determine the metabolic fate of free fatty acids by converting them into activated acyl-CoA derivatives. ACSL family members display distinct substrate preferences and intracellular localization and several genes generate multiple alternatively spliced transcripts [87]. Of particular relevance, ACSL4, which preferentially activates arachidonic and other long-chain PUFAs, undergoes alternative splicing and gives rise to multiple transcript variants [88]. Although ACSL4 has been implicated in the aggressive phenotype of breast cancer cells [89], this evidence does not establish an isoform-specific effect on ferroptosis. Functional studies directly comparing the effects of individual ACSL4 splice variants on ferroptotic sensitivity remain limited [88].

This distinction is important, because the existence of alternatively spliced transcripts does not itself demonstrate that isoform switching determines ferroptosis.

A similarly intriguing situation exists for ACSL3, which promotes utilization of MUFAs and contributes to ferroptosis resistance. Recent pan-cancer analyses have identified multiple ACSL3 transcripts, including a nonsense-mediated mRNA decay (NMD)-associated isoform that appears dysregulated across several tumor types [90]. However, current evidence primarily establishes cancer-associated isoform dysregulation rather than a direct causal relationship between a particular ACSL3 splice variant, MUFA remodeling, and ferroptosis resistance [90]. Thus, ACSL3 illustrates an important knowledge gap: a ferroptosis-relevant lipid enzyme displays alternative transcript usage in cancer, but the functional consequences of this isoform diversity for membrane composition and ferroptotic threshold have not yet been adequately resolved.

#### 5.2.2. Functionally Characterized Lipid-Metabolic Splice Events: NFYA and CPT1A

Alternative splicing may also modify lipid metabolism indirectly through transcriptional regulators that control entire lipogenic programs. A well-defined example is nuclear transcription factor Y subunit alpha (NFYA), which exists as long and short splice variants with distinct biological activities. In triple-negative breast cancer, NFYAv1, the longer isoform, was shown to enhance transcription of the major lipogenic enzymes ACACA (acetyl-CoA carboxylase alpha) and FASN, thereby increasing de novo fatty-acid synthesis and promoting malignant growth. Genetic disruption of the NFYAv1-dependent lipogenic program reduced tumor-cell proliferation and malignant behavior both in vitro and in vivo [91].

Increased “de novo” lipogenesis can support ferroptosis resistance when newly generated saturated fatty acids are converted into MUFAs through SCD1, whereas increased membrane synthesis can also create additional capacity for incorporation of exogenous PUFAs and thereby increase susceptibility under other conditions. The NFYA example therefore emphasizes an important principle: alternative splicing may regulate ferroptosis-relevant lipid metabolism at the level of pathway control rather than exclusively through direct isoform switching of individual enzymes.

Alternative splicing has also been described for carnitine palmitoyltransferase 1A (CPT1A), a key regulator of long-chain fatty-acid entry into mitochondria for β-oxidation. A tumor-associated splice variant, CPT1A variant 2 (CPT1Av2), was identified in breast cancer cells and differs from the canonical CPT1A protein at its C-terminal region. Remarkably, CPT1Av2 lacks classical carnitine palmitoyltransferase activity and instead localizes predominantly to the nucleus, where it interacts with histone deacetylase 1(HDAC 1) and participates in transcriptional regulation of survival and invasion pathways [92]. Silencing CPT1Av2 induced cancer-cell death and altered HDAC-dependent transcriptional programs in breast cancer models. This example demonstrates that alternative splicing can fundamentally redirect the function of a lipid-metabolic enzyme, converting a protein conventionally associated with fatty-acid oxidation into a molecule with a distinct nuclear regulatory role.

The relevance of CPT1A isoform switching to ferroptosis remains indirect at present. Fatty-acid oxidation can influence cellular redox balance, NADPH availability, mitochondrial ROS generation, and the partitioning of fatty acids between oxidation and membrane incorporation, all of which may affect ferroptotic susceptibility. However, there is currently insufficient evidence to conclude that CPT1Av2 directly regulates ferroptosis.

#### 5.2.3. Emerging Candidate Mechanisms in Lipid Storage and Phospholipid Remodeling

Evidence is even more limited for alternative splicing of genes involved in lipid storage and mobilization. Lipid-droplet formation and hydrolysis strongly influence whether PUFAs remain sequestered in neutral lipids or become available for incorporation into membrane phospholipids. Genes such as DGAT1/2, PNPLA2/ATGL, and perilipins are therefore plausible targets through which splicing could alter ferroptosis susceptibility. Alternative PNPLA2 transcripts have been reported in experimental systems, but their physiological and cancer-specific functional significance remains insufficiently established [93]. It would therefore be premature to assign these isoforms a defined role in ferroptosis.

The same caution applies to phospholipid-remodeling enzymes. LPCAT3 is a key determinant of PUFA incorporation into membrane phospholipids, and other lysophospholipid acyltransferases, phospholipases, and lipid-transfer proteins can substantially alter the ferroptosis-sensitive lipidome. Yet, although transcript isoforms exist for several members of these enzyme families, current cancer literature contains relatively few mechanistic studies showing that alternative splicing of these genes directly modifies ferroptotic outcome.

Existing evidence suggests three levels through which alternative splicing may reshape lipid metabolism relevant to ferroptosis. First, lipid-metabolic genes themselves, including members of the ACSL and CPT1 families, generate functionally distinct transcript variants. Second, splice variants of upstream transcriptional regulators such as NFYA can coordinately modify expression of multiple lipogenic enzymes. Third, cancer-associated changes in spliceosome activity may alter broader lipid-metabolic transcript programs even when individual isoforms have not yet been functionally characterized [87,91]. These mechanisms provide a plausible route through which RNA processing could influence the balance between PUFA-rich, ferroptosis-prone membranes and MUFA-enriched, ferroptosis-resistant lipid states.

The current evidence base should not be interpreted as demonstrating that every cancer-associated lipid-metabolic splice event directly controls ferroptosis. The lipid enzymes that determine ferroptosis sensitivity are known, and many of these genes generate multiple transcript isoforms, yet isoform-specific effects on substrate preference, intracellular localization, membrane composition, lipid-droplet dynamics, and ferroptotic response remain poorly characterized. Long-read transcriptomics combined with isoform-specific proteomics, lipidomics, and functional ferroptosis assays will be required to establish which lipid-metabolic splice variants genuinely shift tumor cells between ferroptosis vulnerability and resistance.

### 5.3. Splicing Factors and RNA-Binding Proteins Controlling Ferroptosis

The emerging connection between alternative splicing and ferroptosis is mediated by a relatively small but expanding group of splicing factors and RNA-binding proteins (RBPs) that regulate isoform selection in genes controlling antioxidant defense, iron metabolism, and ferroptotic susceptibility. In cancer, such isoform-specific regulation can shift cells toward either ferroptosis resistance or vulnerability, thereby affecting tumor progression and treatment response [20,94]. Current functional evidence is strongest for hnRNPM, SRSF2, RBFOX2, RNF113A, and TRA2A, each of which has been experimentally linked to ferroptosis through defined alternative-splicing events.

Among heterogeneous nuclear ribonucleoproteins, hnRNPM provides one of the most direct examples of ferroptosis regulation through alternative splicing. In breast cancer, hnRNPM interacts with FMRP and participates in the recognition of splice sites within SLC7A11 pre-mRNA. Increased activity of the FMRP–hnRNPM complex promotes exon skipping and favors production of the SLC7A11-S isoform, which enhances ferroptosis resistance. Functional disruption of hnRNPM reduces formation of this isoform, increases lipid ROS accumulation, and sensitizes breast cancer cells to ferroptotic stress [20].

Cooperative or competitive interactions among RBPs frequently determine exon inclusion or exclusion, allowing cancer cells to integrate signaling, RNA modification, and metabolic state into splice-site selection. RBFOX2, for example, can function together with hnRNPM, heterogeneous nuclear ribonucleoprotein C (hnRNPC), and SRSF1 in distinct binding configurations to regulate alternative splicing outcomes [94].

A second important regulator is SRSF2, a member of the serine/arginine-rich splicing factor family. Direct evidence for its role in ferroptosis has recently been obtained in prostate cancer. CircCNOT6L, which is highly expressed in metastatic prostate cancer, promotes SRSF2-dependent processing of SLC7A11 pre-mRNA, thereby increasing production of a ferroptosis-resistant transcript pattern [81]. Importantly, combining circCNOT6L inhibition with erastin markedly reduced viability in prostate-cancer-derived organoids, demonstrating that the SRSF2-dependent splice program has functional and potentially therapeutic relevance.

SRSF2 is also of particular interest because its activity can be influenced by metabolic and post-translational signals. Recent studies outside the ferroptosis field have shown that metabolic alterations can modify SRSF2 stability and activity through post-translational modifications such as lactylation, thereby altering oncogenic splice patterns [95]. Although this specific mechanism has not yet been directly connected to ferroptosis, it suggests a potentially important route through which tumor metabolism could regulate ferroptosis-related alternative splicing.

RBFOX2 represents another well-characterized splicing regulator with direct functional evidence in ferroptosis. RBFOX2 recognizes specific RNA motifs and regulates both exon inclusion and exclusion depending on the position of its binding sites relative to the regulated exon [96]. In endometrial cancer, circRAPGEF5 was shown to bind RBFOX2 and interfere with its interaction with TFRC pre-mRNA. This interaction promotes exclusion of exon 4 and increases formation of the truncated TFRC-S isoform. Because TFRC-S displays impaired plasma-membrane localization and reduced iron-import capacity, the resulting decrease in the labile iron pool limits lipid-peroxide accumulation and confers ferroptosis resistance. Gain- and loss-of-function experiments demonstrated that manipulating RBFOX2 reverses the ferroptotic effects of circRAPGEF5, establishing a direct circRNA–RBFOX2–TFRC splicing axis controlling iron-dependent ferroptosis [82].

Another direct link between the spliceosome and ferroptosis is provided by RNF113A, an RNA-binding spliceosomal protein overexpressed in lung cancer. Loss of RNF113A enhances cisplatin-induced cell death through several mechanisms, including increased ferroptosis resulting from altered splicing of SAT1 [83]. RNF113A deficiency favors production of the functional SAT1 transcript, increases SAT1 protein expression, enhances ROS generation and lipid oxidative damage, and contributes to ferroptotic cell death. At the same time, RNF113A regulates other survival-associated transcripts such as myeloid cell leukemia 1 (MCL1) and NADPH oxidase activator 1 (NOXA1), demonstrating that a single splicing regulator can coordinate multiple death and stress-response pathways [83].

More recently, TRA2A has emerged as another ferroptosis-regulating splicing factor in ovarian cancer. Gao et al. demonstrated that increased histone H3 lysine 18 lactylation at the TRA2A promoter enhances TRA2A transcription in ovarian cancer cells [85]. Elevated TRA2A subsequently regulates alternative splicing of STIL pre-mRNA, favoring production of the STIL-L isoform. STIL-L alters iron metabolism, suppresses ferroptosis, and decreases sensitivity to cisplatin.

An additional feature emerging from these studies is the frequent involvement of non-coding RNAs and RNA modifications as upstream modulators of splicing factors. CircCNOT6L alters SRSF2-dependent SLC7A11 processing, circRAPGEF5 interferes with RBFOX2-mediated TFRC splicing, and FMRP integrates m6A-associated RNA regulation with hnRNPM-dependent SLC7A11 exon selection [20,81,82]. This suggests that ferroptosis-regulating splicing factors are themselves embedded within larger RNA-regulatory networks. Consequently, the relevant therapeutic target may not always be the splicing factor alone but rather the entire RNA-processing axis that determines production of the ferroptosis-sensitive or ferroptosis-resistant isoform.

Despite these advances, the number of splicing factors with direct experimental validation remains limited. Many serine/arginine-rich splicing proteins (SR proteins), hnRNPs, and other RBPs are strongly dysregulated in cancer and regulate transcripts involved in lipid metabolism, redox control, and stress adaptation, but their direct contribution to ferroptosis has not yet been demonstrated. It is therefore important to distinguish established functional regulators from candidates inferred solely from transcriptomic correlations or ferroptosis-related gene signatures. Future studies combining isoform-resolved RNA sequencing, RBP-binding maps, clustered regularly interspaced short palindromic repeats (CRISPR)-based splicing perturbation, lipidomics, and ferroptosis assays should substantially expand the repertoire of functionally validated regulators.

Current evidence identifies a small but mechanistically diverse group of splicing factors and RBPs that can shift cancer cells between ferroptosis vulnerability and resistance. These findings support the broader concept that the splicing machinery constitutes an active component of the ferroptosis-regulatory network and provide a mechanistic basis for considering RNA-processing factors as potential therapeutic targets in ferroptosis-based cancer strategies.

The functionally validated splicing events currently linking RNA processing to ferroptosis sensitivity or resistance in cancer are summarized in Table 2.

**Figure 2:**
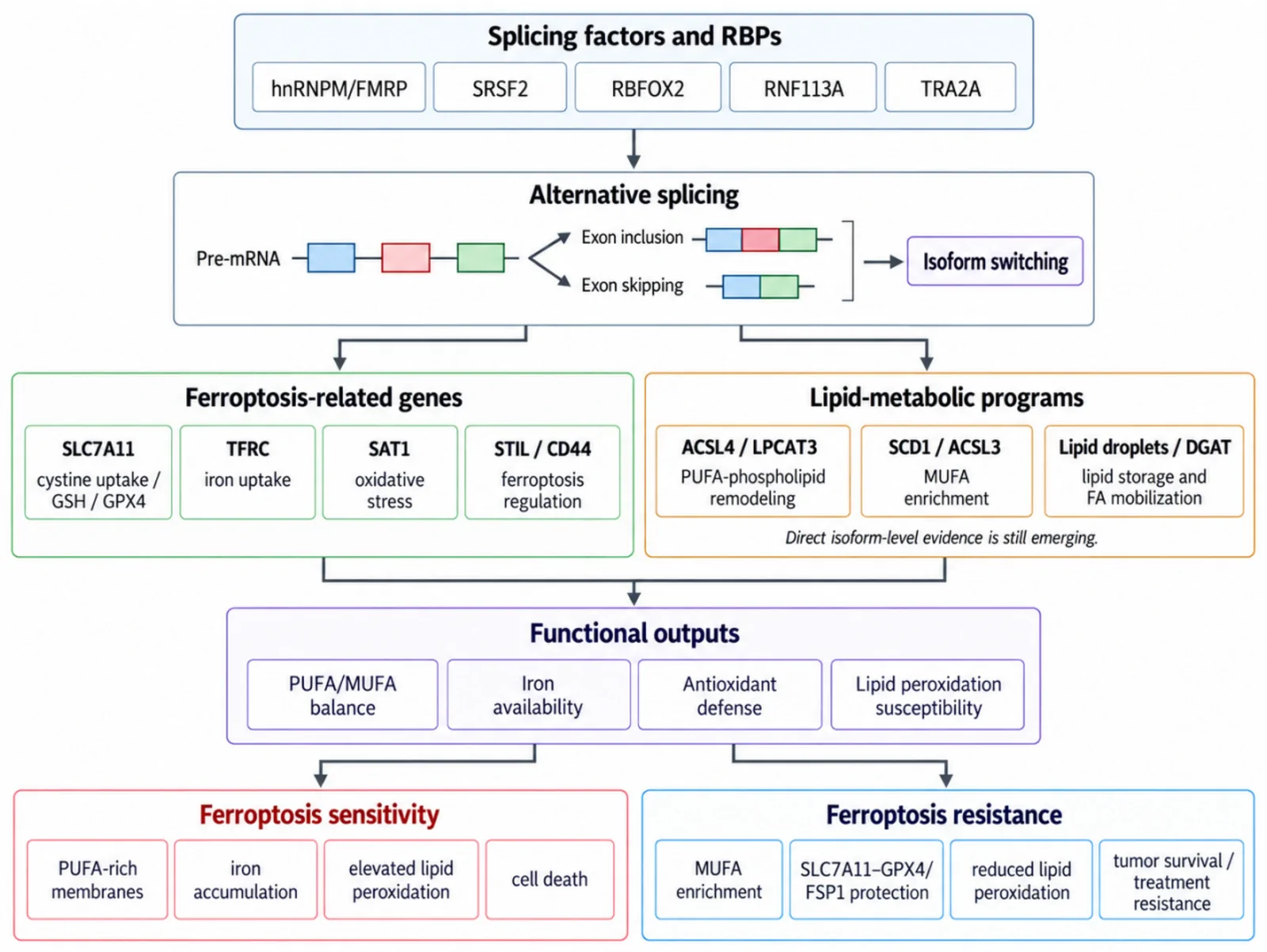
Alternative splicing at the lipid metabolism–ferroptosis interface. Splicing factors and RNA-binding proteins regulate alternative exon selection and isoform switching in genes involved in ferroptosis control and lipid metabolism. Functionally validated events affecting SLC7A11, TFRC, SAT1, STIL, and CD44 can modify cystine uptake, iron availability, oxidative stress, and antioxidant defense, thereby shifting cancer cells toward ferroptosis sensitivity or resistance. In parallel, alternative splicing of lipid-metabolic genes may influence PUFA/MUFA balance, phospholipid remodeling, and lipid storage, although direct isoform-level evidence for these pathways is still emerging. The integrated effects of these RNA-processing events ultimately determine the susceptibility of cancer cells to lipid peroxidation and ferroptotic cell death. Abbreviations: ACSL3, acyl-CoA synthetase long-chain family member 3; ACSL4, acyl-CoA synthetase long-chain family member 4; CD44, cluster of differentiation 44; DGAT, diacylglycerol acyltransferase; FMRP, fragile X messenger ribonucleoprotein; GPX4, glutathione peroxidase 4; GSH, reduced glutathione; hnRNPM, heterogeneous nuclear ribonucleoprotein M; LPCAT3, lysophosphatidylcholine acyltransferase 3; MUFA, monounsaturated fatty acid; PUFA, polyunsaturated fatty acid; RBFOX2, RNA binding fox-1 homolog 2; RBP, RNA-binding protein; RNF113A, ring finger protein 113A; SAT1, spermidine/spermine N1-acetyltransferase 1; SCD1, stearoyl-CoA desaturase 1; SLC7A11, solute carrier family 7 member 11; SRSF2, serine/arginine-rich splicing factor 2; STIL, SCL/TAL1 interrupting locus; TFRC, transferrin receptor; TRA2A, transformer 2 alpha homolog.

The principal alternative-splicing mechanisms linking RNA processing to lipid metabolism and ferroptosis sensitivity or resistance are summarized in Figure 2.

## 6. The Balance Between Ferroptosis Vulnerability and Resistance in Cancer

### 6.1. The Ferroptosis-Sensitive Phenotype

The ferroptosis-sensitive phenotype reflects a metabolic state in which the burden of oxidizable membrane lipids and iron-driven oxidative stress approaches or exceeds the capacity of cellular antioxidant systems to maintain lipid peroxide homeostasis. Rather than being defined by a single molecular marker, ferroptosis sensitivity emerges from the convergence of several features, including PUFA-rich membranes, active ACSL4/LPCAT3-dependent phospholipid remodeling, increased availability of redox-active iron, elevated basal ROS production, and limited antioxidant reserve [6,22,52].

A central characteristic of this phenotype is the enrichment of cellular membranes with PUFA-containing phospholipids. Consequently, cancer cells with a high proportion of oxidation-prone PUFA-phospholipids are metabolically predisposed to ferroptosis because they possess a larger pool of substrates capable of sustaining radical chain reactions [30,31,62].

Experimental suppression of ACSL4 or LPCAT3 reduces PUFA incorporation into membranes and increases resistance to ferroptotic stimuli, supporting their role as important determinants of ferroptosis sensitivity [8,31,65]. However, neither ACSL4 nor LPCAT3 should be regarded as universal stand-alone biomarkers, because their functional impact depends on the broader lipid and antioxidant context of the tumor.

Increased iron uptake, altered ferritin turnover, and changes in iron export can expand the labile Fe^2+^ pool and thereby increase the potential for redox-active iron to participate in oxidative reactions [24,25,53]. This creates an important metabolic paradox: iron is essential for tumor growth, yet the same iron pool can promote lipid peroxidation and ferroptosis when antioxidant protection becomes insufficient. Tumors with increased transferrin receptor activity, enhanced ferritinophagy, or impaired iron sequestration may therefore become particularly vulnerable under ferroptotic stress.

The ferroptosis-sensitive phenotype is also commonly associated with elevated basal oxidative stress. Cancer cells often generate increased ROS as a consequence of oncogenic signaling, mitochondrial activity, inflammatory cues, hypoxia-reoxygenation, and altered redox metabolism [56,58]. At moderate levels, ROS support proliferative and signaling pathways, but persistent oxidative pressure also increases the probability that membrane PUFAs will undergo peroxidation. Cells that already operate close to their oxidative tolerance threshold may therefore be highly sensitive to additional ROS generation or to partial impairment of ferroptosis-defense pathways. Importantly, however, ROS levels alone do not determine ferroptosis susceptibility. A tumor with substantial oxidative stress but predominantly MUFA-rich membranes may remain relatively resistant, whereas a tumor with only moderate ROS but extensive PUFA-phospholipid enrichment may be much more vulnerable. Ferroptosis sensitivity thus reflects the interaction between oxidative pressure and lipid substrate availability rather than either factor in isolation [22,30].

The final component of this phenotype is limited antioxidant reserve relative to oxidative burden. Instead, ferroptosis-sensitive cells may express these systems at substantial levels but remain functionally dependent on them because the rate of lipid-peroxide formation is already high. In such cases, even partial inhibition of one dominant protective pathway can cause a rapid shift toward ferroptotic death [22,42]. This concept is especially relevant for GPX4, because several aggressive or therapy-resistant cancer states exhibit marked dependence on GPX4-mediated lipid peroxide detoxification despite maintaining high expression of other antioxidant pathways [42].

Therapy-resistant and mesenchymal tumor-cell states provide a particularly important example. Viswanathan et al. demonstrated that certain therapy-resistant cancer cells acquire a mesenchymal-like state characterized by enrichment in PUFA-containing lipids and increased dependency on GPX4 for survival [42]. These cells may be resistant to apoptosis-inducing treatments while remaining highly vulnerable to ferroptosis when GPX4 activity is disrupted. From a therapeutic perspective, this is highly relevant because ferroptosis induction may be especially effective in tumors that have already escaped conventional death pathways.

This integrated view is also important for biomarker development. Measuring a single ferroptosis-related molecule is unlikely to capture the full biological state of a tumor. A more informative approach would combine markers of membrane lipid composition, ACSL4/LPCAT3 activity, intracellular iron status, oxidative stress, and antioxidant dependence. Such multidimensional profiling may eventually help distinguish tumors that are intrinsically ferroptosis-prone from those that have acquired robust anti-ferroptotic adaptations. The latter phenotype is discussed in the following section.

### 6.2. The Ferroptosis-Resistant Phenotype

The ferroptosis-resistant phenotype represents a coordinated metabolic and regulatory state in which cancer cells reduce the generation of oxidation-prone membrane lipids while simultaneously strengthening their capacity to neutralize lipid peroxides. Similar to ferroptosis sensitivity, resistance is not determined by a single molecule but emerges from the combined effects of membrane lipid remodeling, cysteine and glutathione metabolism, GPX4-dependent and GPX4-independent antioxidant defenses, and adaptive transcriptional and post-transcriptional programs [6,22,97].

Compared with PUFAs, MUFAs are less susceptible to radical-mediated peroxidation and can therefore reduce the density of highly oxidizable fatty-acyl chains within membrane phospholipids [8,61].

The SCD1–ACSL3 axis plays a major role in establishing this protective lipid state. SCD1 increases endogenous MUFA production, particularly oleate and palmitoleate, whereas ACSL3 activates these fatty acids and facilitates their incorporation into complex lipids [8,9,61]. Several cancer models demonstrate that enhanced SCD1 activity contributes to ferroptosis resistance, while SCD1 inhibition shifts the lipidome toward a more oxidation-prone state and restores susceptibility to ferroptotic stress [9,69].

A second defining feature is reinforcement of the SLC7A11–GSH–GPX4 axis. GSH in turn provides the reducing equivalents required for GPX4-mediated detoxification of phospholipid hydroperoxides [37,41]. Many malignant cells increase SLC7A11 or GPX4 expression during progression or in response to oxidative and therapeutic stress, enabling them to tolerate lipid-peroxidation pressure that would otherwise be lethal. Upregulation of this axis is particularly common in drug-resistant cancer states, where continued GPX4 activity can become essential for survival [41,42].

The robustness of the resistant phenotype is further increased by GPX4-independent defense systems. FSP1 represents the best-characterized alternative pathway and protects cellular membranes by using NAD(P)H to maintain CoQ10 in its reduced, radical-trapping form [44,45]. High FSP1 activity can therefore explain why some tumors remain resistant to GPX4-directed ferroptosis induction and has emerged as an important potential determinant of therapeutic response [44,98]. Complementary pathways involving DHODH–CoQH_2_ and GCH1–BH4 provide additional antioxidant capacity in specific subcellular compartments or metabolic contexts, further increasing redundancy within the ferroptosis-defense network [6,99].

A cancer cell may tolerate inhibition of one ferroptosis-suppressive pathway by increasing reliance on another, thereby preventing lethal lipid-peroxide accumulation. Ferroptosis resistance should therefore be understood as a network property, in which lipid remodeling and antioxidant defense cooperate to maintain membrane integrity [6,22,97].

The FMRP–hnRNPM-dependent generation of the SLC7A11-S isoform in breast cancer provides a particularly clear example, because this splice switch increases glutathione-associated protection and reduces lipid ROS accumulation [20]. Similarly, SRSF2-dependent SLC7A11 processing in prostate cancer, RBFOX2-mediated regulation of TFRC splicing in endometrial cancer, TRA2A-dependent STIL isoform selection in ovarian cancer, and CD44 variant production in lung cancer demonstrate that isoform switching can modify cystine metabolism, iron availability, oxidative stress, or GPX4-related protection [81,82,85,86].

These adaptive splicing programs are especially important because they provide cancer cells with regulatory flexibility. Rather than requiring new mutations or major transcriptional changes, alterations in splice-site selection can rapidly modify the functional output of ferroptosis-related genes in response to oncogenic signaling, metabolic stress, or therapy. Thus, alternative splicing may help consolidate a ferroptosis-resistant phenotype after metabolic or therapeutic selection and could contribute to intertumoral and intratumoral heterogeneity in ferroptosis response.

Importantly, this state is not necessarily permanent. Because many of its components represent acquired metabolic dependencies, ferroptosis resistance may itself create therapeutic liabilities. Simultaneous disruption of membrane-protective lipid remodeling and antioxidant defense, or correction of resistance-promoting splice programs, may therefore shift resistant cancer cells back toward ferroptosis sensitivity.

### 6.3. Biological Consequences

The balance between ferroptosis sensitivity and resistance has important consequences for tumor behavior. Ferroptosis resistance can therefore contribute to tumor persistence and progression, while therapeutic induction of ferroptosis may selectively eliminate malignant populations that have become dependent on lipid-peroxide detoxification [4,6,52]. Recent work increasingly places ferroptosis at the intersection of several difficult-to-treat cancer states, including metastatic progression, cancer stemness, and acquired treatment resistance.

Adaptations that limit lipid peroxidation, including MUFA enrichment and activation of GPX4- or FSP1-dependent defenses, allow cancer cells to tolerate these conditions and maintain proliferative capacity. Conversely, disruption of ferroptosis-defense mechanisms can suppress tumor growth in a variety of experimental models, supporting the concept that resistance to ferroptosis is an active component of malignant-cell survival rather than a passive consequence of altered metabolism [6,100]. This relationship is, however, context-dependent, because ferroptosis can also influence stromal and immune compartments within the tumor microenvironment, making its overall effect on cancer progression more complex than direct tumor-cell killing alone.

Ferroptosis resistance is particularly relevant during metastatic adaptation. Disseminating cancer cells encounter oxidative and metabolic challenges that differ markedly from those in the primary tumor, including detachment from extracellular matrix, exposure to the circulation, colonization of distant tissues, and adaptation to new lipid environments. These transitions can generate substantial lipid oxidative stress, creating strong selective pressure for mechanisms that suppress ferroptosis [9,70]. Experimental evidence showing that ferroptosis induction can inhibit tumor proliferation and metastatic progression supports the view that ferroptosis resistance contributes to metastatic fitness [52].

Lipid availability in distant tissues may further influence this process. This is conceptually important because metastatic adaptation may not simply involve increased resistance to ferroptosis across all conditions; rather, metastatic cells may dynamically modify their ferroptotic threshold according to the metabolic characteristics of the target organ.

The relationship between ferroptosis and cancer stemness is similarly important. Cancer stem cells (CSCs) represent heterogeneous tumor-cell populations with self-renewal capacity, tumor-initiating potential, and pronounced resistance to conventional therapies. Their metabolic and redox characteristics can create distinct ferroptosis dependencies. Some CSC populations exhibit strong antioxidant defenses and increased ability to detoxify lipid peroxides, thereby contributing to ferroptosis resistance and tumor recurrence. In other contexts, however, the metabolic requirements associated with stemness generate specific dependencies on iron, lipid metabolism, or GPX4 that can be therapeutically exploited [101]. Recent analyses therefore emphasize that ferroptosis may provide an opportunity to target CSC populations that otherwise survive chemotherapy or radiotherapy and contribute to disease relapse.

This apparent duality reflects the plasticity of cancer stem-like states. Ferroptosis susceptibility can change during transitions between epithelial, mesenchymal, proliferative, and drug-tolerant phenotypes. Mesenchymal and therapy-persistent states, for example, may accumulate PUFA-containing membrane lipids and become strongly dependent on GPX4-mediated lipid-peroxide detoxification. Such cells can be resistant to apoptosis-inducing therapies yet remain vulnerable to GPX4 inhibition, demonstrating that acquisition of one form of treatment resistance can create a new ferroptotic liability [42].

The strongest translational interest has therefore focused on the relationship between ferroptosis and resistance to anticancer treatment. Chemotherapy, radiotherapy, targeted agents, and immunotherapy can all increase oxidative stress or alter lipid metabolism, thereby potentially increasing ferroptotic pressure. Cancer cells that survive these treatments may adapt by increasing SLC7A11, GPX4, FSP1, MUFA synthesis, or other anti-ferroptotic mechanisms [6,102]. These adaptations can reduce treatment-induced lipid damage and contribute to persistence of resistant clones. Conversely, pharmacological interference with ferroptosis-defense systems has been shown preclinically to resensitize resistant cancer cells to multiple therapeutic modalities.

Radiotherapy provides a particularly relevant example because ionizing radiation generates ROS and can promote lipid peroxidation. Similar principles apply to chemotherapy, where cisplatin-resistant or drug-tolerant states can exploit anti-ferroptotic pathways to survive treatment. Alternative splicing may further stabilize these resistant phenotypes by generating isoforms such as SLC7A11-S, STIL-L, or specific CD44 variants that reduce ferroptotic susceptibility.

Ferroptosis also intersects with immunotherapy resistance. Immune-cell-derived cytokines and cytotoxic signals can alter tumor-cell redox metabolism and potentially promote ferroptotic stress, while tumor cells with reinforced ferroptosis-defense pathways may resist this pressure. Conversely, induction of ferroptosis can modify tumor immunogenicity and influence macrophages, T cells, and other components of the tumor microenvironment. Preclinical evidence suggests that ferroptosis modulation may enhance responses to immune checkpoint blockade and other immune-based strategies, although these effects are complex and highly context-dependent [103].

Conversely, these same adaptations can create dependencies on lipid-remodeling and antioxidant-defense pathways that may be therapeutically exploitable. This duality explains why ferroptosis is increasingly viewed not simply as another death mechanism, but as a metabolic pressure point that can shape tumor evolution and therapeutic response. At the same time, its translational exploitation remains largely preclinical, and ferroptosis-directed therapies have not yet entered routine clinical testing, underscoring the need for reliable biomarkers and rational patient selection.

## 7. Therapeutic Exploitation of the Splicing–Lipid Metabolism–Ferroptosis Axis

### 7.1. Targeting Ferroptosis Defense Mechanisms

Therapeutic induction of ferroptosis can be achieved by weakening the antioxidant systems that prevent lethal lipid-peroxide accumulation. Among these, SLC7A11, GPX4, and FSP1 represent the most relevant intervention points because they protect cancer cells through complementary mechanisms [4,6,104].

Erastin provided the initial proof of principle for this strategy, while other agents, including sulfasalazine and sorafenib, can interfere with system Xc^−^, although they are not selective SLC7A11 inhibitors [1,37,38,43]. Tumors strongly dependent on extracellular cystine may therefore be particularly susceptible, whereas activation of compensatory cysteine-producing pathways can limit therapeutic efficacy. Moreover, the existence of resistance-associated SLC7A11 splice variants suggests that isoform composition may eventually complement total SLC7A11 expression as a predictor of response.

Experimental inhibitors such as RAS-selective lethal 3 (RSL3), ML162, and ML210 have demonstrated strong antitumor activity in preclinical systems, particularly in mesenchymal and therapy-resistant cancer-cell states characterized by GPX4 dependency [42,105]. Nevertheless, limited pharmacokinetics, systemic toxicity, and the essential role of GPX4 in normal tissues remain important barriers. Targeted delivery systems and protein-degradation strategies are therefore being explored to improve tumor selectivity [100,106].

Genetic or pharmacological interference with FSP1 can restore ferroptosis sensitivity, and recent in vivo evidence supports its therapeutic relevance across several cancer models [107]. These findings suggest that combined inhibition of GPX4 and FSP1, or simultaneous weakening of SLC7A11-dependent glutathione metabolism and downstream antioxidant defenses, may overcome compensatory resistance.

Beyond classical experimental ferroptosis inducers, several compounds with anticancer activity have been shown to engage ferroptosis-related pathways in preclinical models. In hepatocellular carcinoma, dihydroartemisinin promoted ferroptosis by inhibiting the ATF4–SLC7A11 axis and enhanced sensitivity to sorafenib. In breast cancer, tanshinone IIA reduced SLC7A11 stability through suppression of KDM1A–PIAS4-mediated SUMOylation, thereby promoting ferroptosis and limiting tumor progression. Similarly, Mori Folium ethanol extracts induced ferroptosis in gastric cancer by modulating the AKT–GSK3β–NRF2 pathway and enhanced cisplatin sensitivity. In colorectal cancer, angelic acid promoted NRF2 degradation and increased sensitivity to sulfasalazine-induced ferroptosis. Although these findings expand the range of potential therapeutic strategies beyond direct ferroptosis inducers, their clinical applicability remains to be established [108,109,110,111].

Overall, SLC7A11, GPX4, and FSP1 represent complementary therapeutic nodes. Their successful exploitation will likely require tumor-specific assessment of antioxidant dependency, compensatory pathways, and splice-isoform composition to maximize efficacy while limiting toxicity.

### 7.2. Targeting Lipid Metabolism to Promote Ferroptosis

Therapeutic approaches may increase PUFA-containing phospholipids, reduce protective MUFA remodeling, or redirect fatty acids from storage toward membrane incorporation [8,64,77].

ACSL4 is a major pro-ferroptotic determinant because it activates long-chain PUFAs that subsequently enter membrane phospholipids [31,64]. Therefore, therapeutic exploitation of ACSL4 differs from classical enzyme inhibition: preservation or enhancement of ACSL4-dependent PUFA remodeling would be expected to increase ferroptosis susceptibility, whereas ACSL4 loss favors resistance. Because selective ACSL4 activators are not currently established, indirect strategies may involve increasing appropriate PUFA substrates or blocking pathways that compete with ACSL4-dependent lipid remodeling.

In contrast, SCD1 is a more conventional therapeutic target. By generating MUFAs, SCD1 creates a membrane environment less susceptible to peroxidation; its inhibition can increase lipid ROS and restore ferroptosis sensitivity in several tumor models [9,69]. ACSL3 similarly promotes MUFA utilization, and its inhibition may weaken this protective membrane-remodeling program [61,64].

Sequestration of PUFAs into LDs can reduce their incorporation into peroxidation-prone membranes, whereas inhibition of DGAT-dependent neutral-lipid synthesis may redirect PUFAs toward membrane phospholipids and enhance ferroptotic susceptibility [77]. However, the effects of lipid-droplet manipulation remain context-dependent.

An especially attractive strategy is simultaneous enhancement of pro-ferroptotic lipid remodeling and inhibition of protective MUFA pathways. In pancreatic ductal adenocarcinoma models, CD147 inhibition increased ACSL4 while suppressing the sterol regulatory element-binding protein 1 (SREBP1)–SCD1 axis, shifting the PUFA/MUFA balance toward ferroptosis and enhancing gemcitabine efficacy [112].

### 7.3. Targeting Alternative Splicing

Targeting alternative splicing offers a distinct therapeutic opportunity because it intervenes upstream of the protein networks that establish ferroptosis resistance. Three main approaches are relevant: modulation of splicing factors, splice-switching oligonucleotides, and isoform-specific targeting [113,114,115].

Small-molecule modulation of spliceosome components or splicing-regulatory kinases such as serine/arginine protein kinase (SRPKs) and CDC-like kinase (CLKs) has shown antitumor potential, although broad inhibition of RNA processing may produce considerable toxicity [113,114,115,116]. More selective strategies are therefore attractive. In breast cancer, disruption of the FMRP–hnRNPM axis reduces production of the ferroptosis-resistant SLC7A11-S isoform and increases lipid peroxidation [20]. Similarly, SRSF2-dependent SLC7A11 processing in prostate cancer, RBFOX2-dependent TFRC splicing in endometrial cancer, and TRA2A-mediated STIL splicing in ovarian cancer identify additional RNA-processing networks that could potentially be targeted to restore ferroptosis susceptibility [81,82,85].

A more precise approach involves splice-switching antisense oligonucleotides (SSOs). These molecules bind defined pre-mRNA sequences and modify exon inclusion or exclusion without globally suppressing the spliceosome [114,117]. Conceptually, an SSO preventing formation of SLC7A11-S could favor a more ferroptosis-sensitive transcript profile, while correction of TFRC exon skipping could restore functional transferrin-receptor expression and intracellular iron uptake. Similar strategies could potentially target STIL-L or resistance-associated CD44 variants. At present, however, ferroptosis-directed SSOs targeting these specific events remain experimental rather than clinically validated.

Isoform-specific targeting provides another level of selectivity. Aberrant transcripts frequently contain unique exon–exon junctions that can be targeted using antisense-mediated degradation, siRNA, RNA interference, or related approaches [114,118]. Selective depletion of SLC7A11-S, TFRC-S, or STIL-L could theoretically reverse ferroptosis resistance while preserving the canonical transcript. Such strategies may be particularly valuable when complete inhibition of the corresponding gene would produce unacceptable toxicity.

Splicing-directed approaches may also be combined with established ferroptosis inducers. Correcting a resistance-promoting splice event may lower the ferroptotic threshold rather than inducing cell death by itself. The combination of circCNOT6L inhibition with erastin in prostate-cancer-derived organoids provides early support for this concept [81].

Important limitations remain, including tumor delivery, off-target splice alterations, and the highly context-dependent functions of individual splicing factors. Moreover, many ferroptosis-associated isoforms have been validated in only one tumor type. Nevertheless, the high molecular specificity of splice-switching and isoform-selective strategies makes them particularly attractive for future precision ferroptosis therapy.

### 7.4. Combination Strategies and Biomarkers

Given the substantial heterogeneity of ferroptosis sensitivity among tumors, ferroptosis-directed interventions are likely to be most effective when combined with established anticancer therapies and guided by molecular biomarkers [6,100].

Preclinical studies have shown that disruption of SLC7A11, GPX4, or protective lipid-remodeling pathways can enhance responses to agents such as cisplatin and gemcitabine [6,104]. Similarly, radiotherapy generates ROS and lipid oxidative damage, providing a rationale for combining irradiation with inhibition of ferroptosis-defense pathways [119]. Targeted therapies may also create secondary dependencies on GPX4, SLC7A11, FSP1, or MUFA-producing pathways, exposing drug-tolerant persister cells to ferroptosis-based interventions.

The interaction with immunotherapy is particularly interesting. Activated CD8^+^ T cells can enhance tumor-cell ferroptosis through interferon-γ-mediated effects on cystine metabolism, and higher ferroptosis-related activity has been associated with improved outcomes in some immunotherapy-treated cohorts [120]. However, ferroptosis in immune cells may impair antitumor immunity, emphasizing the importance of tumor-selective induction.

Single markers such as ACSL4, SLC7A11, GPX4, or FSP1 are unlikely to capture the full ferroptotic state. More informative models should integrate membrane lipid composition, PUFA/MUFA balance, iron status, oxidative stress, and antioxidant dependence [22,100].

Lipidomic profiling may provide a particularly direct measure of susceptibility because ferroptosis ultimately depends on membrane lipid composition. Clinical lipidomic studies have already demonstrated that circulating lipid signatures can predict therapeutic response in cancer, supporting the feasibility of this approach [121].

For the present framework, splice-isoform signatures are equally important. Gene-level expression may overlook biologically relevant differences when sensitive and resistant tumors produce different isoforms of the same gene. Ferroptosis-related alternative-splicing signatures have already shown associations with prognosis and predicted treatment response in gastric cancer [122], although prospective validation remains necessary.

Ultimately, the most informative approach may combine lipidomic, antioxidant, and splice-isoform data into a composite ferroptosis vulnerability signature. This integrated strategy may improve patient selection and help identify the therapeutic combinations most likely to exploit ferroptosis successfully.

**Figure 3:**
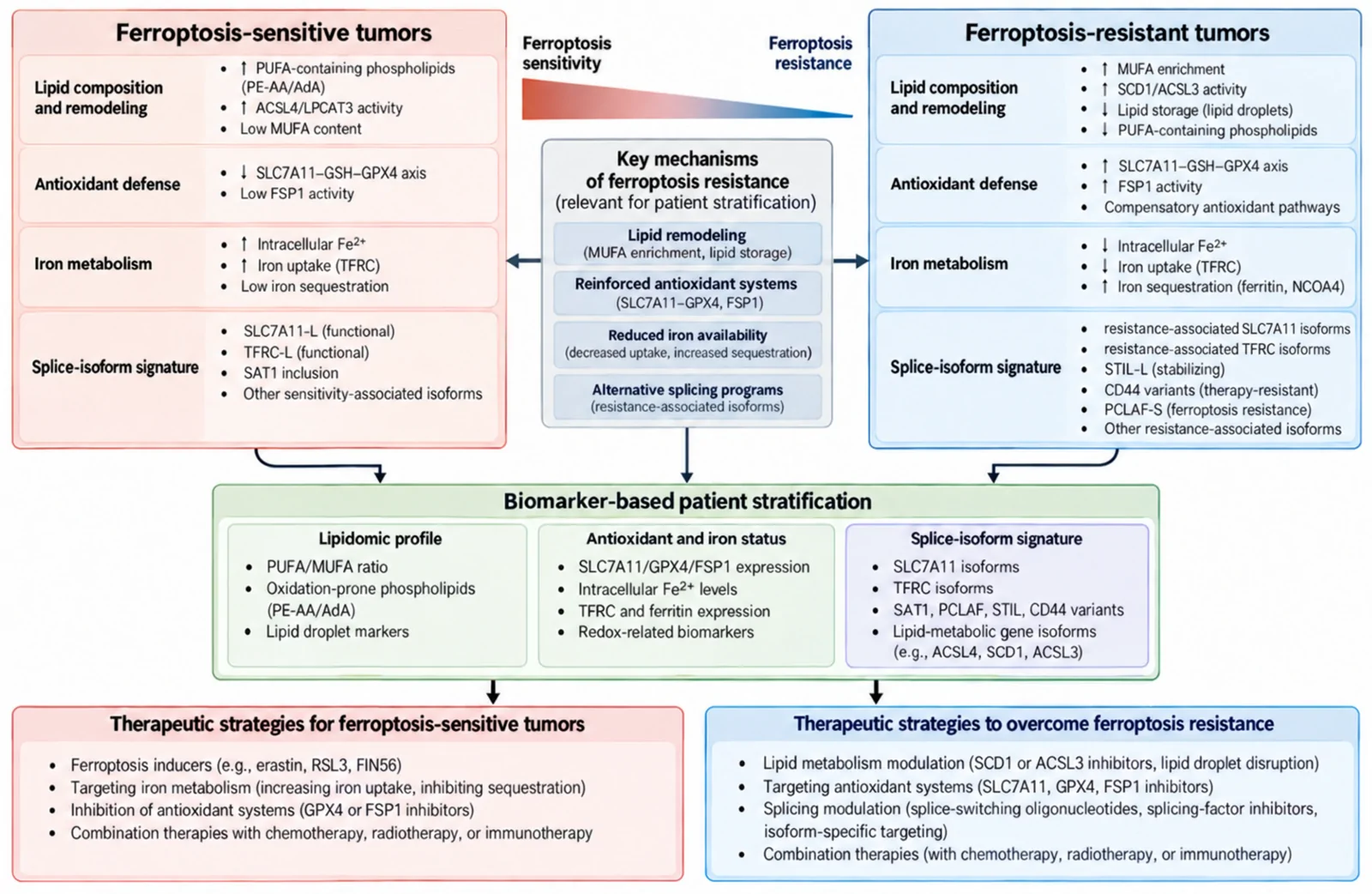
Molecular mechanisms of ferroptosis sensitivity and resistance in cancer: implications for biomarker-based patient stratification and therapeutic targeting. The figure illustrates the molecular and metabolic characteristics associated with ferroptosis-sensitive and ferroptosis-resistant tumor phenotypes. Ferroptosis sensitivity is associated with PUFA-enriched phospholipids, increased ACSL4/LPCAT3 activity, elevated intracellular iron availability, reduced antioxidant defenses, and sensitivity-associated splice isoforms. In contrast, ferroptosis resistance is favored by MUFA enrichment, SCD1/ACSL3-dependent lipid remodeling, enhanced antioxidant protection through the SLC7A11–GSH–GPX4 and FSP1 pathways, reduced iron availability, and resistance-associated alternative-splicing programs. These mechanisms provide a potential basis for future patient stratification through the integration of lipidomic profiles, antioxidant and iron-related biomarkers, and splice-isoform signatures. Such multidimensional profiling may help identify ferroptosis-vulnerable tumors and guide therapeutic approaches aimed at overcoming ferroptosis resistance, including modulation of lipid metabolism, antioxidant defenses, alternative splicing, and combination treatments. The proposed framework remains exploratory and requires further experimental and clinical validation. Abbreviations: AA, arachidonic acid; ACSL3, acyl-CoA synthetase long-chain family member 3; ACSL4, acyl-CoA synthetase long-chain family member 4; AdA, adrenic acid; CoQ, coenzyme Q; FSP1, ferroptosis suppressor protein 1; GPX4, glutathione peroxidase 4; GSH, glutathione; LPCAT3, lysophosphatidylcholine acyltransferase 3; MUFA, monounsaturated fatty acid; NCOA4, nuclear receptor coactivator 4; PE, phosphatidylethanolamine; PUFA, polyunsaturated fatty acid; SAT1, spermidine/spermine N1-acetyltransferase 1; SCD1, stearoyl-CoA desaturase 1; SLC7A11, solute carrier family 7 member 11; STIL, SCL/TAL1 interrupting locus; TFRC, transferrin receptor; PCLAF, PCNA clamp-associated factor; RSL3, RAS-selective lethal 3.

The major molecular mechanisms underlying ferroptosis sensitivity and resistance, together with their potential implications for biomarker-based patient stratification and therapeutic targeting, are summarized in Figure 3.

### 7.5. Cancer-Specific Experimental and Clinical Evidence: Therapeutic Response and Prognostic Implications

The clinical relevance of the splicing–lipid metabolism–ferroptosis axis varies according to tumor origin, molecular characteristics, and therapeutic exposure. Although several alternative-splicing events have been functionally linked to ferroptosis resistance, their translational significance is supported by heterogeneous evidence derived from cancer cell lines, animal models, patient-derived organoids, clinical specimens, and retrospective survival analyses. Importantly, the experimental demonstration of ferroptosis regulation through specific splice isoforms must be distinguished from the clinical validation of prognostic or predictive biomarkers. Available studies suggest cancer-specific associations with tumor aggressiveness, metastatic progression, treatment resistance, and patient survival, although the strength of evidence differs considerably among malignancies.

#### 7.5.1. Breast Cancer

The FMRP–hnRNPM–SLC7A11 regulatory axis provides a relevant example of the relationship between alternative splicing, ferroptosis resistance, and tumor progression. Wang et al. demonstrated that FMRP promotes the generation of the SLC7A11-S isoform through interaction with hnRNPM, thereby reducing lipid peroxidation and supporting malignant-cell survival. Experimental disruption of this pathway increased ferroptotic susceptibility and suppressed tumor growth. The clinical component of the study included 60 patients with breast cancer and demonstrated increased expression of FMRP, hnRNPM, and SLC7A11 in tumor tissues. Higher FMRP expression was associated with unfavorable clinical outcomes, while SLC7A11 and hnRNPM were also overexpressed in tumor specimens. However, the reported survival analyses involved different endpoints, and the association for hnRNPM was not statistically significant. Furthermore, these findings do not establish the independent prognostic value of the SLC7A11-S isoform.

These findings support the clinical relevance of the regulatory axis but do not yet demonstrate that isoform-specific expression predicts chemotherapy response or overall survival independently of other established prognostic factors [20].

#### 7.5.2. Prostate Cancer

The circCNOT6L–SRSF2–SLC7A11 pathway has been associated with ferroptosis resistance and metastatic progression. Liu et al. identified increased circCNOT6L expression in prostate cancer, particularly in metastatic tissues, and demonstrated that suppression of this regulatory axis reduced cancer-cell proliferation, migration, and invasion. These findings were supported by xenograft experiments showing reduced tumor growth and metastatic dissemination. Importantly, the combination of circCNOT6L inhibition and erastin significantly reduced the viability of prostate cancer-derived organoids, providing experimental evidence for therapeutic sensitization. Human tissue analyses further demonstrated associations between the regulatory network and aggressive tumor characteristics, while transcriptomic analyses linked SRSF2 and SLC7A11 expression to unfavorable progression-related outcomes. However, progression-free interval associations should not be interpreted as evidence of reduced overall survival, and the clinical predictive value of individual SLC7A11 splice isoforms remains unestablished. Thus, the available evidence supports a role in tumor aggressiveness and suggests a potential therapeutic vulnerability rather than a clinically validated strategy for overcoming treatment resistance [81].

#### 7.5.3. Endometrial Cancer

The circRAPGEF5–RBFOX2–TFRC pathway illustrates the contribution of alternative splicing to ferroptosis resistance through altered cellular iron availability. Zhang et al. examined 30 endometrial cancer specimens and 25 normal endometrial tissue samples and identified increased circRAPGEF5 expression in malignant tissues. Higher expression was significantly associated with FIGO stage (*p* = 0.026) and histological grade (*p* = 0.005), supporting a relationship with tumor aggressiveness. Mechanistic experiments demonstrated that circRAPGEF5 interferes with RBFOX2-dependent processing of TFRC pre-mRNA, promotes exon exclusion, and reduces intracellular iron uptake and lipid peroxidation. Functional studies in cell-based and animal models confirmed its tumor-promoting effects. Nevertheless, the study did not establish a direct association between the TFRC splice variant and overall survival or demonstrate a clinically relevant improvement in chemotherapy response following modulation of this pathway. Therefore, its significance is currently supported primarily by mechanistic evidence and clinicopathological associations rather than validated clinical outcomes [82].

#### 7.5.4. Lung Cancer

Two experimentally characterized splicing-dependent pathways illustrate distinct relationships between ferroptosis regulation, treatment resistance, and prognosis. First, Shostak et al. demonstrated that RNF113A is overexpressed in pulmonary adenocarcinoma tissues and that higher expression is associated with shorter patient survival. In experimental models, RNF113A depletion increased susceptibility to cisplatin-induced cell death through multiple mechanisms, including enhanced production of functional SAT1 transcripts, increased ferroptotic activity, and altered regulation of apoptosis-associated proteins. These findings suggest that RNF113A contributes to treatment resistance through broader spliceosome-dependent survival programs rather than exclusively through ferroptosis [83]. Second, Pan et al. identified a MEN1-dependent mechanism controlling CD44 alternative splicing and ferroptosis susceptibility. Experimental loss of MEN1 increased the production of CD44 variant isoforms, reduced lipid peroxidation, and promoted tumor growth and metastatic behavior, whereas interference with CD44v6 restored ferroptotic susceptibility and suppressed malignant progression. Importantly, analysis of a cohort comprising 516 patients with lung adenocarcinoma demonstrated that increased CD44 percent-spliced-in values, reflecting higher variant inclusion, were associated with poorer overall survival, advanced tumor stage, and metastasis. In contrast, total CD44 mRNA expression showed inconsistent prognostic associations across datasets. This distinction provides particularly relevant clinical support for isoform-resolved analysis, suggesting that splice-variant composition may convey prognostic information not captured by conventional gene-level measurements. Nevertheless, its predictive value for response to specific anticancer treatments requires prospective validation [86].

#### 7.5.5. Hepatocellular Carcinoma

The SRSF2–PCLAF pathway provides an example of alternative-splicing-dependent resistance to targeted therapy in hepatitis B virus-associated hepatocellular carcinoma. Liu et al. reported increased PCLAF expression in HBV-related tumors and identified elevated PCLAF tv1 expression in clinical specimens. Mechanistic studies demonstrated that HBV-mediated suppression of SRSF2 promotes abnormal PCLAF splicing, reduces intracellular Fe^2+^ availability, and increases GPX4 expression, thereby limiting ferroptotic activity. Functional experiments further showed that this pathway contributes to decreased sensitivity to sorafenib and that modulation of the SRSF2–PCLAF tv1 axis can alter therapeutic susceptibility. Although these findings support a biologically plausible mechanism of acquired resistance, the available clinical data do not establish a direct association between PCLAF tv1 expression and overall survival or prospectively demonstrate its ability to predict sorafenib response. Consequently, this mechanism represents a potential therapeutic target requiring further clinical investigation [84].

#### 7.5.6. Ovarian Cancer

The H3K18la–TRA2A–STIL pathway illustrates how metabolic and epigenetic alterations can influence ferroptosis through alternative splicing. Gao et al. demonstrated that increased histone H3 lysine 18 lactylation enhances TRA2A expression and favors production of the STIL-L isoform, which suppresses ferroptosis by modifying iron metabolism. Functional experiments showed that TRA2A depletion increased ferroptotic susceptibility and enhanced cisplatin sensitivity, including in xenograft models. Although these findings support a contribution to platinum resistance, the independent prognostic significance of STIL-L and its association with overall survival remain insufficiently established [85].

Additional clinical evidence supports the relevance of ferroptosis-associated antioxidant and lipid-metabolic pathways in ovarian cancer, although these findings do not directly validate the TRA2A–STIL splice event. In a retrospective cohort of 192 patients with epithelial ovarian cancer, Wu et al. reported that high co-expression of SLC7A11 and GPX4 was strongly associated with platinum resistance (RR = 60.46, 95% CI: 22.76–160.58) and independently predicted shorter overall survival (HR = 4.44, 95% CI: 2.77–7.14) and progression-free survival (HR = 5.73, 95% CI: 3.86–8.73). Complementary experiments showed that inhibition of SLC7A11 and GPX4 reduced resistance in ovarian cancer cells [123]. More recently, Yu et al. investigated the combined expression of GPAT4, a lipid-metabolic enzyme, and SLC7A11 in epithelial ovarian cancer. Their clinical analysis included 102 patients and demonstrated an association between elevated expression of both proteins, platinum resistance, and reduced overall survival. In experimental models, combined inhibition of GPAT4 and SLC7A11 enhanced the cytotoxic effects of cisplatin in platinum-resistant ovarian cancer cells [124]. Collectively, these studies provide clinical evidence linking ferroptosis-associated metabolic pathways with chemotherapy response and prognosis. However, these associations are observational and cannot establish that therapeutic induction of ferroptosis improves survival or that alternative-splicing events directly determine clinical outcomes.

#### 7.5.7. Pancreatic Ductal Adenocarcinoma

Evidence from lipid-metabolic targeting further supports the therapeutic relevance of ferroptosis regulation independently of direct splice-isoform modulation. Li et al. demonstrated that pharmacological targeting of CD147 using dracorhodin perchlorate promotes ferroptosis by increasing ACSL4 expression and suppressing SREBP1–SCD1 signaling, thereby shifting lipid composition toward a more peroxidation-prone state. The combination with gemcitabine enhanced antitumor activity in pancreatic cancer cells and several complementary preclinical models, including orthotopic xenografts, genetically engineered KPC mice, patient-derived organoids, and patient-derived xenografts. The consistency of these observations across different experimental systems strengthens their biological relevance and supports further therapeutic investigation. Nevertheless, responses observed in patient-derived models do not establish clinical efficacy, and no prospective evidence currently demonstrates that this strategy improves chemotherapy response or overall survival in patients with pancreatic cancer [112].

#### 7.5.8. Gastric Cancer

Evidence for the potential prognostic value of ferroptosis-related alternative splicing has also emerged from transcriptomic analyses. Long et al. developed a risk model incorporating 12 ferroptosis-related alternative-splicing events and evaluated its association with clinical outcomes in 406 patients with gastric cancer. Patients classified into the high-risk group exhibited significantly poorer overall survival, while multivariable analysis identified the risk score as an independent prognostic factor (HR = 1.461, 95% CI: 1.345–1.587). The model yielded areas under the receiver operating characteristic curve of 0.739, 0.723, and 0.753 for predicting survival at 1, 3, and 5 years, respectively. Additional analyses identified differences in immune-cell infiltration and computationally predicted treatment sensitivity between risk categories, while functional experiments suggested that SF3A2 contributes to ferroptosis resistance and malignant-cell proliferation. These findings support the potential utility of alternative-splicing signatures for prognostic stratification. However, computational predictions of drug response do not constitute evidence of clinical treatment efficacy, and the proposed signature requires external and prospective validation before implementation in clinical practice [122].

Current evidence suggests that alternative splicing and lipid-metabolic regulation influence tumor aggressiveness, metastasis, treatment resistance, and prognosis in a cancer-specific manner. However, functional evidence derives mainly from cellular and animal models, while clinical studies largely report associations with gene expression, clinicopathological features, and survival. The prognostic relevance of individual splice isoforms remains insufficiently established, and experimental improvements in drug sensitivity have not yet translated into demonstrated survival benefits in patients. Future studies integrating isoform-resolved transcriptomics, lipidomics, functional ferroptosis assays, and clinical outcomes are needed to validate predictive biomarkers and assess the therapeutic potential of this regulatory axis.

The available cancer-specific experimental and clinical evidence linking ferroptosis-related alternative splicing and lipid-metabolic pathways to therapeutic response, tumor aggressiveness, and prognosis is summarized in Table 3.

## 8. Conclusions and Future Perspectives

Ferroptosis is increasingly recognized as a metabolically driven vulnerability in cancer, determined by the interplay between lipid composition, iron availability, oxidative pressure, and antioxidant defense.

Emerging evidence indicates that alternative splicing adds another regulatory layer to this balance. Functionally validated splice events involving SLC7A11, TFRC, SAT1, PCLAF, STIL, and CD44 demonstrate that isoform selection can modify cystine transport, iron availability, antioxidant capacity, and ferroptotic susceptibility independently of total gene expression.

However, the direct contribution of alternative splicing to lipid-metabolic control of ferroptosis remains insufficiently characterized.

Future studies should therefore prioritize identification of biologically relevant splice isoforms and distinguish them from non-functional transcript variants. This will require integration of long-read RNA sequencing, proteomics, lipidomics, and functional ferroptosis assays. Such approaches could determine whether specific isoforms alter PUFA/MUFA balance, membrane phospholipid composition, antioxidant dependency, or ferroptotic threshold and thereby establish causal links between RNA processing and lipid-dependent cell death.

Tumor heterogeneity must also be considered, because distinct cellular populations may differ substantially in lipid composition, antioxidant dependencies, and splicing programs. Isoform-resolved single-cell and spatial approaches may therefore help identify ferroptosis-resistant subpopulations, including stem-like, therapy-persistent, or metastatic cells.

From a translational perspective, the ultimate objective is to develop ferroptosis vulnerability signatures that integrate lipidomic, redox, and splice-isoform information. This multidimensional approach may improve patient selection and help identify the most appropriate therapeutic combinations.

Overall, alternative splicing appears to contribute to the metabolic plasticity that determines ferroptosis sensitivity and resistance in cancer. Integrating isoform-level RNA analysis with lipidomic and functional profiling may reveal previously unrecognized vulnerabilities and provide a more precise framework for developing ferroptosis-based anticancer strategies.

## Figures and Tables

**Table 1:** Lipid metabolic determinants of ferroptosis sensitivity and resistance in cancer.

Lipid Metabolic Factor	Main Metabolic Function	Effect on Cellular Lipid Composition	Effect on Ferroptosis	Cancer-Related Significance	References
**ACSL4**	Activates long-chain PUFAs, particularly AA and AdA, generating PUFA-CoA derivatives	Increases PUFA incorporation into membrane phospholipids and enriches oxidation-prone lipid species	Promotes sensitivity	High ACSL4 activity can create a ferroptosis-prone lipidome and increase susceptibility to ferroptotic stress	[30,31,63,64]
**LPCAT3**	Reacylates lysophospholipids using PUFA-CoA substrates	Promotes incorporation of PUFAs into membrane phospholipids	Promotes sensitivity	Supports formation of peroxidation-prone phospholipids; reduced LPCAT3 activity can attenuate ferroptosis	[62,65]
**PUFA-containing phospholipids**	Provide major substrates for lipid peroxidation	Increase abundance of highly oxidizable membrane phospholipids	Promote sensitivity	AA and AdA-containing phospholipids are prominent substrates during ferroptotic execution	[3,30,62]
**SCD1**	Converts saturated fatty acids into MUFAs	Increases oleate/palmitoleate and enriches membranes in more oxidation-resistant lipid species	Promotes resistance	Frequently supports tumor survival, metabolic adaptation and resistance to ferroptotic stress	[68,69,70,71]
**ACSL3**	Activates MUFAs and facilitates their incorporation into complex lipids	Favors MUFA-containing phospholipids and decreases relative PUFA-driven membrane oxidation	Promotes resistance	Required for MUFA-mediated protection from ferroptosis in several cancer models	[61,70,72]
**MUFA enrichment**	Reduces the proportion of highly peroxidizable fatty-acyl chains in membranes	Replaces/dilutes PUFA-containing phospholipids	Promotes resistance	Can protect tumor cells from ferroptotic stress induced by GPX4 inhibition or system Xc^−^ blockade	[8,61,67]
**De novo lipogenesis**	Generates fatty acids for membrane synthesis and storage	Can increase saturated fatty-acid and MUFA pools; may also support membrane expansion	Context-dependent	May promote resistance when coupled to SCD1-mediated MUFA production, but can also increase substrates available for subsequent PUFA remodeling	[39,59,60,74]
**Lipid droplets**	Store fatty acids as neutral lipids, mainly triacylglycerols and cholesteryl esters	Sequester fatty acids away from membrane phospholipids	Usually protective, context-dependent	PUFA sequestration can reduce lipid peroxidation; mobilization of stored PUFAs may instead restore ferroptosis sensitivity	[8,10,75,76,77,78]
**DGAT-dependent neutral-lipid synthesis**	Esterifies fatty acids into triacylglycerols for lipid-droplet storage	Diverts PUFAs from membrane phospholipids toward neutral-lipid pools	Promotes resistance	DGAT inhibition can redistribute PUFAs toward membrane phospholipids and increase ferroptotic susceptibility	[77]
**Lipolysis/lipophagy**	Mobilizes fatty acids from lipid droplets	Releases stored fatty acids for oxidation or membrane reincorporation	Context-dependent	Release of PUFA-rich stores may increase substrates for phospholipid peroxidation and promote ferroptosis	[8,75,78]
**Phospholipid remodeling**	Controls exchange and incorporation of fatty-acyl chains into membrane phospholipids	Determines PUFA/MUFA distribution within cellular membranes	Bidirectional	Central determinant of whether the lipidome becomes ferroptosis-prone or ferroptosis-resistant	[36,62,64,79]
**POR and lipid-oxidizing enzymes**	Facilitate phospholipid oxidation	Increase formation of oxidized phospholipid species	Promote sensitivity	Contribute to lipid-peroxide generation and amplification of ferroptotic damage	[32]
**Tumor-stroma lipid exchange**	Modifies extracellular lipid supply and phospholipid composition	Can reshape tumor-cell membrane lipid profile	Context-dependent	May alter ferroptosis susceptibility through changes in phospholipid remodeling and lipid availability	[80]

Abbreviations: AA, arachidonic acid; ACSL3, acyl-CoA synthetase long-chain family member 3; ACSL4, acyl-CoA synthetase long-chain family member 4; AdA, adrenic acid; DGAT, diacylglycerol acyltransferase; LPCAT3, lysophosphatidylcholine acyltransferase 3; MUFA, monounsaturated fatty acid; POR, cytochrome P450 oxidoreductase; PUFA, polyunsaturated fatty acid; SCD1, stearoyl-CoA desaturase 1.

**Table 2:** Functionally validated alternative splicing events regulating ferroptosis in cancer.

Splicing Regulator/RBP	Target Gene	Alternative Splicing Event/Isoform	Cancer Type	Main Functional Consequence	Effect on Ferroptosis	Biological/Therapeutic Relevance	References
FMRP/hnRNPM	SLC7A11	Exon-skipping event generating SLC7A11-S	Breast cancer	SLC7A11-S supports GSH homeostasis and reduces lipid ROS accumulation	Resistance	Promotes tumor growth and is associated with poorer prognosis; disruption of the FMRP–hnRNPM–SLC7A11 axis restores ferroptotic susceptibility	[20]
SRSF2, regulated by circCNOT6L	SLC7A11	SRSF2-dependent differential processing of SLC7A11 pre-mRNA	Prostate cancer	Maintains SLC7A11 expression, increases GSH and limits Fe^2+^/lipid ROS accumulation	Resistance	Promotes invasion and metastasis; circCNOT6L silencing combined with erastin suppresses prostate-cancer organoids	[81]
RBFOX2, modulated by circRAPGEF5	TFRC	Exon 4 skipping, generating truncated TFRC-S	Endometrial cancer	Impaired membrane localization of TFRC, reduced iron uptake and decreased labile iron pool	Resistance	Promotes endometrial cancer proliferation; targeting circRAPGEF5–RBFOX2 may restore ferroptotic sensitivity	[82]
RNF113A	SAT1	Regulation of exon 4 inclusion/skipping; exon 4 skipping generates the functional SAT1-coding transcript	Lung cancer cells exposed to cisplatin	Increased functional SAT1 promotes oxidative stress and lipid ROS accumulation	Sensitivity/induction	RNF113A depletion enhances cisplatin-induced ferroptotic cell death, linking spliceosome activity to chemotherapy response	[83]
SRSF2 (downregulated by HBV)	PCLAF	Alternative splicing of exon 3, favoring PCLAF tv1	HBV-associated hepatocellular carcinoma	PCLAF tv1 decreases intracellular Fe^2+^ and increases GPX4 expression	Resistance	Promotes sorafenib resistance; restoring SRSF2 or disrupting the PCLAF tv1 axis restores ferroptotic sensitivity	[84]
TRA2A	STIL	Isoform switching favoring STIL-L	Ovarian cancer	Alters iron metabolism and reduces ferroptotic stress	Resistance	Contributes to cisplatin resistance; TRA2A depletion increases ferroptosis and treatment sensitivity	[85]
MEN1–PAF1/RNA Pol II elongation axis	CD44	Increased inclusion of variable exons, particularly CD44v6-containing isoforms, after MEN1 loss	Lung cancer	CD44 variants increase GPX4-associated protection and reduce cytosolic and lipid ROS	Resistance	CD44v6 interference restores ferroptosis and suppresses tumor growth and metastasis in MEN1-deficient tumors	[86]

Abbreviations: FMRP, fragile X messenger ribonucleoprotein; GSH, glutathione; GPX4, glutathione peroxidase 4; hnRNPM, heterogeneous nuclear ribonucleoprotein M; RBP, RNA-binding protein; ROS, reactive oxygen species; SRSF2, serine/arginine-rich splicing factor 2; RBFOX2, RNA binding fox-1 homolog 2; SAT1, spermidine/spermine N1-acetyltransferase 1; SLC7A11, solute carrier family 7 member 11; TFRC, transferrin receptor; TRA2A, transformer 2 alpha homolog.

**Table 3:** Cancer-specific experimental and clinical evidence linking ferroptosis-related alternative splicing and lipid-metabolic pathways to therapeutic response and prognosis.

Study/Cancer Type	Study Model	Investigated Pathway	Main Findings
**Wang et al., 2024** [20] **Breast cancer**	Cells; animal models; human specimens (*n* = 60)	FMRP–hnRNPM–SLC7A11-S	Ferroptosis resistance, tumor progression, and prognostic associations
**Liu et al., 2025** [81] **Prostate cancer**	Cells; xenografts; organoids; human specimens	circCNOT6L–SRSF2–SLC7A11	Metastasis; erastin sensitization after pathway inhibition; progression-free interval associations
**Zhang et al., 2022** [82] **Endometrial cancer**	Cells; animal models; human specimens	circRAPGEF5–RBFOX2–TFRC	Ferroptosis resistance; association with tumor stage and grade
**Shostak et al., 2020** [83] **Lung cancer**	Cells; experimental models; human tumor data	RNF113A–SAT1	Experimental cisplatin resistance; adverse survival association
**Liu et al., 2023** [84] **Hepatocellular carcinoma**	Cells; human specimens	SRSF2–PCLAF tv1	Reduced ferroptosis and experimental sorafenib resistance
**Gao et al., 2025** [85] **Ovarian cancer**	Cells; xenografts; human specimens	TRA2A–STIL-L	Ferroptosis resistance and reduced cisplatin sensitivity
**Pan et al., 2026** [86] **Lung adenocarcinoma**	Cells; animal models; human cohort	MEN1–CD44	Tumor growth, metastasis, and unfavorable overall survival associated with variant inclusion
**Li et al., 2025** [112] **Pancreatic cancer**	Cells; multiple animal and patient-derived models	CD147–ACSL4/SREBP1–SCD1	Enhanced gemcitabine efficacy in preclinical models
**Long et al., 2024** [122] **Gastric cancer**	Patient transcriptomic cohort; cell experiments	Ferroptosis-related AS signature	Overall-survival association; computational predictions of treatment response
**Wu et al., 2022** [123] **Ovarian cancer**	Clinical cohort; cell experiments	SLC7A11–GPX4	Platinum resistance and unfavorable overall and progression-free survival
**Yu et al., 2025** [124] **Ovarian cancer**	Clinical cohort; cell experiments	GPAT4–SLC7A11	Platinum resistance and unfavorable overall survival

Note. Studies [112,123,124] investigate ferroptosis-associated metabolic or antioxidant pathways rather than functionally validated alternative-splicing events. Refs. [123,124] are provisional citation labels pending final reference renumbering. Abbreviations: ACSL4, acyl-CoA synthetase long-chain family member 4; AS, alternative splicing; CD44, cluster of differentiation 44; CD147, cluster of differentiation 147 (basigin); circCNOT6L, circular RNA derived from CNOT6L (CCR4-NOT transcription complex subunit 6-like); circRAPGEF5, circular RNA derived from RAPGEF5 (Rap guanine nucleotide exchange factor 5); FMRP, fragile X messenger ribonucleoprotein; GPAT4, glycerol-3-phosphate acyltransferase 4; GPX4, glutathione peroxidase 4; H3K18la, histone H3 lysine 18 lactylation; HBV, hepatitis B virus; hnRNPM, heterogeneous nuclear ribonucleoprotein M; MEN1, menin 1; OS, overall survival; PCLAF, PCNA clamp-associated factor; PCNA, proliferating cell nuclear antigen; PFI, progression-free interval; PFS, progression-free survival; RBFOX2, RNA-binding fox-1 homolog 2; RNF113A, ring finger protein 113A; SAT1, spermidine/spermine N1-acetyltransferase 1; SCD1, stearoyl-CoA desaturase 1; SF3A2, splicing factor 3a subunit 2; SLC7A11, solute carrier family 7 member 11; SREBP1, sterol regulatory element-binding protein 1; SRSF2, serine/arginine-rich splicing factor 2; STIL, SCL/TAL1 interrupting locus protein; TFRC, transferrin receptor; TRA2A, transformer 2 alpha homolog.

## Data Availability

No new data were created or analyzed in this study. Data sharing is not applicable to this article.

## Abbreviations

The following abbreviations are used in this manuscript:

AA	arachidonic acid
ACACA	acetyl-CoA carboxylase alpha
ACSL	acyl-CoA synthetase long-chain family
ACSL3	acyl-CoA synthetase long-chain family member 3
ACSL4	acyl-CoA synthetase long-chain family member 4
AdA	adrenic acid
AIFM2	apoptosis-inducing factor mitochondria-associated 2
AS	alternative splicing
ATGL	adipose triglyceride lipase
ATP	adenosine triphosphate
BH4	tetrahydrobiopterin
CD44	cluster of differentiation 44
CD44v6	CD44 variant 6
CD147	cluster of differentiation 147
circCNOT6L	circular RNA derived from CNOT6L
CNOT6L	CCR4-NOT transcription complex subunit 6-like
circRAPGEF5	circular RNA derived from RAPGEF5
RAPGEF5	Rap guanine nucleotide exchange factor 5
CLK	CDC-like kinase
CoQ10	coenzyme Q10
CoQH2	reduced coenzyme Q (ubiquinol)
CPT1A	carnitine palmitoyltransferase 1A
CRISPR	clustered regularly interspaced short palindromic repeats
CSC	cancer stem cell
DGAT	diacylglycerol acyltransferase
DHODH	dihydroorotate dehydrogenase
DNA	deoxyribonucleic acid
FASN	fatty acid synthase
FMRP	fragile X messenger ribonucleoprotein
FSP1	ferroptosis suppressor protein 1
GCH1	GTP cyclohydrolase 1
GPAT4	glycerol-3-phosphate acyltransferase 4
GPX4	glutathione peroxidase 4
GSH	reduced glutathione
GSSG	oxidized glutathione
GTP	guanosine triphosphate
H3K18la	histone H3 lysine 18 lactylation
HBV	hepatitis B virus
HDAC	histone deacetylase
hnRNPC	heterogeneous nuclear ribonucleoprotein C
hnRNPM	heterogeneous nuclear ribonucleoprotein M
LPCAT3	lysophosphatidylcholine acyltransferase 3
MCL1	myeloid cell leukemia 1
MEN1	menin 1
MLKL	mixed lineage kinase domain-like protein
MUFA	monounsaturated fatty acid
NAD(P)H	reduced nicotinamide adenine dinucleotide (phosphate)
NADPH	reduced nicotinamide adenine dinucleotide phosphate
NFYA	nuclear transcription factor Y subunit alpha
NMD	nonsense-mediated mRNA decay
NOXA1	NADPH oxidase activator 1
NRF2	nuclear factor erythroid 2-related factor 2
OS	overal survival
PAF1	polymerase-associated factor 1
PCLAF	PCNA clamp-associated factor
PCNA	proliferating cell nuclear antigen
PFI	progression-free interval
PFS	progression-free survival
PL-OH	phospholipid alcohol
PL-OOH	phospholipid hydroperoxide
PNPLA2	patatin-like phospholipase domain-containing protein 2
POR	cytochrome P450 oxidoreductase
PUFA	polyunsaturated fatty acid
RBP	RNA-binding protein
RBFOX2	RNA binding fox-1 homolog 2
RIPK3	receptor-interacting serine/threonine-protein kinase 3
RNA	ribonucleic acid
RNF113A	ring finger protein 113A
ROS	reactive oxygen species
RSL3	RAS-selective lethal 3
SAT1	spermidine/spermine N1-acetyltransferase 1
SCD1	stearoyl-CoA desaturase 1
SF3A2	splicing factor 3a subunit 2
SLC3A2	solute carrier family 3 member 2
SLC7A11	solute carrier family 7 member 11
SLC7A11-S	short splice isoform of SLC7A11
SR proteins	serine/arginine-rich splicing proteins
SREBP1	sterol regulatory element-binding protein 1
SRPK	serine/arginine protein kinase
SRSF2	serine/arginine-rich splicing factor 2
SSO	splice-switching oligonucleotide
STIL	SCL/TAL1 interrupting locus
STIL-L	long isoform of STIL
system Xc^−^	cystine/glutamate antiporter system Xc^−^
TFRC	transferrin receptor
TFRC-S	short/truncated splice isoform of TFRC
TRA2A	transformer 2 alpha homolog
